# Hyperspectral Image Classification Based on an Improved Octopus Optimization Algorithm

**DOI:** 10.3390/biomimetics11080542

**Published:** 2026-08-03

**Authors:** Yong Xu, Libo Jiang, Yi Zhang

**Affiliations:** College of Electrical and Computer Science, Jilin Jianzhu University, Changchun 130119, China; xuyong@jlju.edu.cn (Y.X.); jianglibo@student.jlju.edu.cn (L.J.)

**Keywords:** improving Octopus Optimization Algorithm, swarm intelligence optimization algorithms, hyperspectral image, image classification, hyperparameter optimization

## Abstract

This paper proposes a multi-strategy-enhanced Octopus Optimization Algorithm (OOA) for hyperparameter optimization in hyperspectral image classification. Hyperspectral images pose significant challenges due to their numerous spectral bands, high dimensionality, and complex spectral differences between classes, which complicate classification modeling. The classification performance of support vector machine (SVM) classifiers is also highly dependent on parameter settings. The original OOA is extended by incorporating an initialization strategy based on elite backpropagation, a multi-stage nonlinear adaptive parameter control mechanism, an elite-guided differential mutation strategy, a Lévy flight restart mechanism with stagnation monitoring, and a stable boundary handling strategy. These enhancements constitute the IOOA-SVM parameter optimization framework. The proposed method is evaluated against OOA, Particle Swarm Optimization (PSO), Sand Cat Swarm Optimization (SCSO), Salp Swarm Algorithm (SSA), Grey Wolf Optimizer (GWO), Arithmetic Optimization Algorithm (AOA), Differential Evolution (DE) and Linear Population Size Reduction Success-History Based Adaptive Differential Evolution (L-SHADE) on the CEC2017 test set, achieving superior results on most of the 29 test functions, IOOA achieved the best results on average for 27 of the 29 test functions, outperforming the original OOA on all 29 test functions and demonstrating superior performance on most stability metrics. Different improvement strategies yield varying degrees of performance gains for the algorithm; among them, the elite-guided differential mutation strategy produces the most significant performance improvement. The synergy and complementarity among multiple strategies play a major role in enhancing the performance of the Improved Octopus Optimization Algorithm. Experimental results show that the SVM classifier optimized using the improved OOA achieves a classification accuracy of 97.3731%, representing a 0.2278 percentage point improvement over the original algorithm and demonstrating strong overall optimization performance.

## 1. Introduction

Hyperspectral image classification represents a central task in remote sensing data processing. Hyperspectral images possess a large number of spectral bands, high spectral resolution, and substantial information content. Despite these advantages, they are challenged by high dimensionality, significant data redundancy, spectral similarity between classes, and a limited number of labeled samples. As a result, classification models are highly sensitive to parameter tuning [1,2]. Support vector machines (SVMs) have received considerable attention in hyperspectral image classification due to their strong generalization capabilities and effectiveness with small datasets. Melgani and Bruzzone demonstrated the performance of SVMs in high-dimensional hyperspectral remote sensing classification, highlighting their suitability for small sample sizes [3]. However, the classification performance of SVMs is highly dependent on the appropriate configuration of penalty and kernel parameters. Therefore, achieving efficient, stable automatic hyperparameter optimization across a complex parameter space is a critical challenge for improving hyperspectral image classification performance [4]. Classifier parameter tuning in this context constitutes a complex optimization problem. The objective functions in such problems often exhibit nonlinearity, multiple local optima, high dimensionality, and black-box characteristics. Consequently, traditional methods such as analytical approaches, gradient-based techniques, and exhaustive search often suffer from significant limitations in computational efficiency, global search capability, and applicability. In contrast, meta-heuristic optimization algorithms do not require gradient information from the objective function. As a result, they provide greater generality, flexibility, and global optimization capabilities, and have become essential tools for addressing complex optimization problems [5].

Meta-heuristic optimization algorithms have become essential tools for automatically optimizing classifier parameters in hyperspectral image classification. Ding et al. applied the Particle Swarm Optimization (PSO) algorithm to optimize support vector machine (SVM) classification of hyperspectral images [6]. Samadzadegan et al. employed the ant colony optimization algorithm to address parameter determination in SVM classification of hyperspectral images [7]. Zhao et al. improved the artificial bee colony algorithm to search for SVM penalty and kernel parameters [8]. Zhu et al. compared the optimization performance of various swarm intelligence algorithms in SVM classification of hyperspectral images. Their findings revealed significant differences among optimizers regarding convergence speed, parameter stability, feature selection capability, and classification accuracy, demonstrating that the choice of optimizer directly influences hyperspectral performance [9]. However, existing performance methods continue to face challenges, including low search accuracy, significant result variability, and limited improvements in high-precision performance across complex parameter spaces. Therefore, continuing to explore optimization methods with novel search mechanisms and potential for improvement, building on existing optimization algorithms, remains of great significance for enhancing the effectiveness of SVM parameter optimization in hyperspectral image classification. Compared with optimization algorithms such as PSO [10], GWO [11], SSA [12], AOA [13], SCSO [14], DE [15], and L-SHADE [16], the Octopus Optimization Algorithm (OOA) [17] is a relatively new swarm intelligence optimization algorithm and it has not yet been fully explored for its application in the task of optimizing classification parameters for hyperspectral images. The OOA search mechanism incorporates both global search and local exploration processes, making it well-suited for the continuous, multi-peaked search characteristics of RBF-SVM penalty and kernel parameters. This paper therefore adopts OOA as the basis for its research and explores its potential applications in the task of hyperparameter optimization for hyperspectral image classification.

The Octopus Optimization Algorithm (OOA), introduced by Kaiguang Wang et al. in 2026 [17], employs a swarm search mechanism inspired by octopus behavior, including prey search, predator evasion, attack, and mating. This approach has been applied to engineering problems involving continuous optimization. While the algorithm has demonstrated effectiveness, several limitations remain when addressing complex numerical optimization and engineering parameter tuning. First, random initialization often leads to an uneven distribution of the initial population, reducing early search quality. Second, position updates primarily depend on predefined rules, limiting the ability to dynamically adjust search intensity during different iteration stages and making it challenging to balance exploration and exploitation. Finally, as iterations progress, population diversity decreases. Due to the lack of effective perturbation and restart mechanisms, the algorithm is susceptible to becoming trapped in local optima when solving complex multi-modal problems, thereby reducing convergence accuracy and operational stability in later stages. These limitations have affected the practical effectiveness of OOA in complex numerical optimization and engineering parameter tuning.

This paper presents an improved OOA framework for the SVM hyperparameter optimization task in hyperspectral image classification, addressing the shortcomings of the original algorithm, and proposes an Improved Octopus Optimization Algorithm (IOOA) based on a comprehensive strategy. This algorithm enhances the original OOA by improving population initialization quality, coordination between exploration and exploitation, avoidance of local optima, solution precision, and operational stability. First, this paper introduces an initialization strategy based on elite backpropagation [18] to enhance the diversity of the initial population and the coverage of the search space. Second, this paper designs a multi-stage, nonlinear, adaptive parameter control mechanism [19,20] to enhance the algorithm’s ability to dynamically adjust the intensities of global search and local exploration at different iteration stages. Furthermore, this paper incorporates the elite-guided differential variation strategy [21,22] into the position update process to enhance local exploration and fine-grained search capabilities in the later stages. Finally, this paper incorporates the Lévy flight restart mechanism [23] based on stagnation monitoring [24,25] and the stable boundary handling strategy [26] to enhance the algorithm’s ability to escape local optima and maintain search robustness. Different strategies contribute to improvements in algorithm performance to varying degrees; among them, the elite-guided differential mutation strategy yields the most significant performance gains. The synergistic and complementary effects of these various strategies are the source of IOOA’s overall performance advantage. In addition, this paper systematically evaluates the algorithm’s performance on the CEC2017 test dataset and applies it to optimize RBF-SVM hyperparameters in hyperspectral image classification, thereby verifying the effectiveness of the improved algorithm and its practical application.

The main contributions of this paper are as follows:

1. This paper proposes an Improved Octopus Optimization Algorithm (IOOA) to address the shortcomings of the original OOA in terms of initial population quality, search balance, and the ability to avoid local optima.
2. A framework for collaborative improvement through multiple strategies has been developed. The innovation of this paper lies in the mechanistic, collaborative design of elite-guided backward learning initialization, nonlinear adaptive parameter control, elite-guided differential mutation, and Lévy flight restart with stagnation monitoring and stable boundary handling based on the shortcomings of different search phases in OOA and the requirements of hyperspectral image classification. This forms a collaborative improvement framework to enhance the algorithm’s global exploration, local exploration, and search robustness.
3. The optimized performance of IOOA was verified through comparative experiments using multiple algorithms on the CEC2017 test function set.
4. IOOA was applied to the optimization of RBF-SVM hyperparameters for hyperspectral image classification at Pavia University to validate its practical value.

The rest of the article is organized as follows: Section 2 reviews related work, including SVM classifiers, the fundamental principles of the original OOA, and the developed IOOA-SVM parameter-optimization framework. Section 3 details the design methodology for the improved IOOA algorithm and presents its flowchart. Section 4 reports the CEC2017 function set experiments and analyzes the results. Quantitative analysis demonstrates that, compared to other algorithms, IOOA achieves superior convergence accuracy, faster convergence speed, and greater stability. Section 5 explores the application of IOOA for hyperparameter tuning in hyperspectral image classification and compares its performance with other algorithms, confirming its superiority. The final chapter summarizes the paper and outlines future research directions.

## 2. Related Works

### 2.1. Support Vector Machine (SVM)

Support vector machines (SVMs), as supervised learning methods, construct optimal classification hyperplanes within the feature space. SVMs maximize the margin between classes, thereby enhancing the model’s generalization by minimizing structural risk. Their effectiveness with small sample sizes and robust generalization make SVMs particularly suitable for hyperspectral image classification, which is characterized by high dimensionality, numerous spectral bands, limited labeled samples, and complex spectral differences between categories. As a result, SVMs are among the most widely used classifiers for hyperspectral image classification [27]. In practical classification tasks, samples are often not linearly separable. The soft margin mechanism in SVMs permits some classification errors and employs the penalty parameter C to balance the trade-off between the classification margin and the cost of misclassification. A larger penalty parameter enforces stricter classification accuracy on the training data but may reduce generalization. In contrast, a smaller penalty parameter increases tolerance for misclassifications, potentially improving generalization but possibly decreasing accuracy and causing underfitting. SVMs frequently employ kernel functions to map original data into high-dimensional feature spaces, thereby transforming nonlinear classification problems into linear ones. The radial basis function (RBF) kernel, which is well-suited for nonlinear data and features a relatively simple parameter structure, is particularly effective at capturing the complex spectral distribution of hyperspectral images. Consequently, RBF kernels are widely used in hyperspectral image classification tasks [28]. The RBF kernel parameter γ determines the influence range of individual samples. An excessively large γ can make the model overly sensitive to local variations, resulting in overfitting, while an excessively small γ produces overly smooth classification boundaries, thereby reducing classification accuracy.

The classification performance of radial basis function-based support vector machines (RBF-SVMs) is highly dependent on the settings of the regularization parameter C and the kernel parameter γ [29]. Traditional methods, such as experience-based tuning and grid search, exhibit low search efficiency, high computational cost, and strong dependence on the parameter range. These limitations hinder the reliable identification of optimal or near-optimal parameter combinations, particularly for complex, high-dimensional problems. An Improved Octopus Optimization Algorithm is introduced for hyperparameter tuning of the penalty parameter C and the kernel parameter γ, aiming to automatically identify superior parameter combinations and deliver enhanced classification models for subsequent hyperspectral image classification tasks.

The parameters are encoded in logarithmic space to improve search stability and enhance comparability across different orders of magnitude, as follows:

(1) $$x=\left[x_{1},x_{2}\right]=\left[{log}_{10}C,{log}_{10}\gamma \right]$$

Accordingly, the parameter search range is set to

(2) $$x_{1}\in \left[-2,4\right],\;\;\;\;x_{2}\in \left[-4,4\right]$$

That is,

(3) $$C\in \left[10^{-2},10^{4}\right],\;\;\;\;\gamma \in \left[10^{-4},10^{4}\right]$$

### 2.2. The Octopus Optimization Algorithm

The Octopus Optimization Algorithm (OOA), introduced by Wang et al. in 2026 [17], is a swarm intelligence optimization method that models the biological behaviors of octopuses, including foraging, predator avoidance, hunting, and mating. OOA employs a specialized population-based search strategy that balances global exploration with local exploitation, making it particularly effective for continuous, complex optimization problems.

#### 2.2.1. Population Initialization

The octopus population is initialized based on the boundaries of the search space. The mathematical expression for population initialization is as follows:

(4) $$x_{i,j}=l_{j}+rand\cdot \left(u_{j}-l_{j}\right)$$

In the equation, $x_{i,j}$ represents the position of the i-th octopus individual in the j-th dimension; rand is a random number in the range [0, 1]; and $u_{j}$ and $l_{j}$ represent the upper and lower bounds of the j-th dimension, respectively.

In the Octopus Optimization algorithm, the octopus population can be represented by the following matrix:

(5) $$X={\left[\begin{array}{c} X_{1}\\ \begin{array}{c} \vdots \\ X_{i}\\ \vdots \end{array}\\ X_{N} \end{array}\right]}_{N\times D}={\left[\begin{array}{ccc} \begin{array}{c} x_{1,1}\\ \vdots \\ \begin{array}{c} x_{i,1}\\ \vdots \\ x_{N,1} \end{array} \end{array} & \begin{array}{c} \cdots \\ \ddots \\ \begin{array}{c} \cdots \\ \ddots \\ \cdots \end{array} \end{array} & \begin{array}{ccc} \begin{array}{c} x_{1,j}\\ \vdots \\ \begin{array}{c} x_{i,j}\\ \vdots \\ x_{N,j} \end{array} \end{array} & \begin{array}{c} \cdots \\ \ddots \\ \begin{array}{c} \cdots \\ \ddots \\ \cdots \end{array} \end{array} & \begin{array}{c} x_{1,D}\\ \vdots \\ \begin{array}{c} x_{i,D}\\ \vdots \\ x_{N,D} \end{array} \end{array} \end{array} \end{array}\right]}_{N\times D}$$

In the equation, X represents the octopus population location matrix; $X_{i}$ represents the location of the i-th octopus; N represents the population size; and D represents the problem dimension.

Each individual’s objective function value in the population can be expressed using the following formula:

(6) $$F={\left[\begin{array}{c} \begin{array}{c} F_{1}\\ \vdots \end{array}\\ \begin{array}{c} F_{i}\\ \vdots \end{array}\\ F_{N} \end{array}\right]}_{N\times 1}={\left[\begin{array}{c} f\left(X_{1}\right)\\ \begin{array}{c} \vdots \\ f\left(X_{i}\right)\\ \vdots \end{array}\\ f\left(X_{N}\right) \end{array}\right]}_{N\times 1}$$

In this equation, F represents the population fitness vector; $F_{i}$ represents the objective function value of the i-th individual; and $f\left(\cdot \right)$ denotes the objective function. The population initialization phase provides the foundation for updating the positions of the octopus individuals and searching for the optimal solution.

#### 2.2.2. Potential Prey Search and Predator Avoidance Phases

In the Octopus Optimization Algorithm (OOA), global search is primarily executed during the potential prey search and predator avoidance phases. The potential prey search phase models the octopus’s environmental perception, enabling individuals to conduct an extensive search for promising regions within the search space. The squid uses jet propulsion to perform rapid jumps, broadening the search range and improving the population’s capacity to escape local optima during the predator-avoidance phase. Collectively, these two processes comprise the algorithm’s global search phase.

The octopus uses acute environmental awareness to identify potential prey and move toward regions with a higher likelihood of prey during the prey-searching phase. This mechanism increases the population’s coverage of the search space and improves the algorithm’s exploratory capability. The velocity for this phase is calculated as follows:

(7) $$V_{i}^{t+1}=FV_{i}^{t}+A_{1}e^{-\beta_{2}\sqrt{r_{0}}\frac{t}{T}}\left(X_{k_{0}}^{t}-X_{i}^{t}\right)+A_{3}e^{-\beta_{1}\sqrt{r_{ibest}}\left(1-\sqrt{\frac{t}{T}}\right)}\left(X_{ibest}^{t}-X_{i}^{t}\right)$$

The corresponding position update formula is:

(8) $$X_{i}^{t+1}=X_{i}^{t}+V_{i}^{t+1}$$

Second, during the predator evasion phase, the octopus’s jet propulsion mechanism allows it to quickly change direction and escape. In the algorithm, this is represented by a global jump search method that incorporates a recoil mechanism, with the velocity update formula as follows:

(9) $$V_{i}^{t+1}=FV_{i}^{t}+A_{1}e^{-\beta_{2}\sqrt{r_{1}}\frac{t}{T}}\left(X_{k_{1}}^{t}-X_{i}^{t}\right)+A_{1}e^{-\beta_{2}\sqrt{r_{2}}\frac{t}{T}}\left(X_{k_{2}}^{t}-X_{i}^{t}\right)$$

The corresponding position update formula is as follows:

(10) $$X_{i}^{t+1}=X_{i}^{t}+V_{i}^{t+1}+\left|A\right|\frac{{\left(V_{i}^{t}\right)}^{2}-{\left(V_{i}^{t-1}\right)}^{2}}{2al},\left|A\right|>1$$

In this equation, $V_{i}^{t}$ and $V_{i}^{t+1}$ represent the velocity of the i-th individual at the t-th and (t + 1)-th iterations, respectively; F is the fluid resistance term; A, $A_{1}$, and $A_{3}$ are stochastic control parameters; $\beta_{1}$ and $\beta_{2}$ are perception coefficients; $X_{ibest}^{t}$ is the individual’s historical best position; $X_{k_{0}}^{t}$, $X_{k_{1}}^{t}$, and $X_{k_{2}}^{t}$ denote reference positions in stochastic directions; $r_{0}$, $r_{1}$, $r_{2}$, and $r_{ibest}$ represent the distances from the current individual to the corresponding positions; al is the recoil acceleration term; T is the maximum number of iterations; and t is the current iteration number.

#### 2.2.3. Prey Attack and Mating Update Phases

Local evolution in the OOA is primarily driven by predation and mating. Upon detecting prey, an octopus moves toward the optimal region, guided by both its individual optimal position and the global optimal position. This approach enhances local search capabilities. Subsequent mating updates recombine locally optimal individuals to generate new solutions, enabling a more detailed search in the vicinity of the optimal region. Collectively, these processes constitute the local development phase of the algorithm.

The octopus transitions from exploring a broad area to focusing on prime hunting grounds during the prey-hunting phase. This process enhances the population’s ability to explore regions near local optima, thereby improving the algorithm’s convergence accuracy. The velocity is calculated as follows:

(11) $$\begin{array}{c} V_{i}^{t+1}=FV_{i}^{t}+A_{3}e^{-\beta_{1}\sqrt{r_{ibest}}\left(1-\sqrt{\frac{t}{T}}\right)}\left(X_{ibest}^{t}-X_{i}^{t}\right)\\ \;\;\;\;\;\;\;\;+A_{2}e^{-\beta_{3}\sqrt{r_{gbest}}\left(1-\frac{t}{T}\right)}\left(X_{gbest}^{t}-X_{i}^{t}\right) \end{array}$$

The position update formula is as follows:

(12) $$X_{i}^{t+1}=X_{i}^{t}+V_{i}^{t+1}+\left|A\right|\frac{{\left(V_{i}^{t}\right)}^{2}-{\left(V_{i}^{t-1}\right)}^{2}}{2al},\left|A\right|\leq 1$$

Second, in addition to simulating predatory behavior, OOA also mimics the mating behavior of octopuses to further enhance the algorithm’s local search capabilities. Depending on the distance between male and female individuals, the mating renewal formula can be expressed as follows:

(13) $$X_{i}^{M}=\left\{\begin{array}{c} p\cdot K_{1}^{t}+\left(1-p\right)\cdot K_{2}^{t},0<R\leq pl\\ rand\cdot K_{2}^{t},R>pl\\ rand\cdot K_{2}^{t}+K_{1}^{t},R=0 \end{array}\right.$$

In the equation, $X_{i}^{M}$ represents the candidate positions generated by the i-th individual during the mating update phase; $K_{1}^{t}$ and $K_{2}^{t}$ denote the positions of the male and female individuals, respectively; p is a random coefficient; R is the distance between the male and female individuals; and pl is the threshold for the safe mating distance.

This algorithm uses a greedy selection strategy to select new positions generated during the mating and update phase, thereby ensuring that the population continuously converges toward a more optimal solution, that is,

(14) $$X_{i}^{t+1}=\left\{\begin{array}{c} X_{i}^{M},F_{i}^{M}<F_{i}\\ X_{i}^{t},else \end{array}\right.$$

Here, $F_{i}^{M}$ represents the objective function value corresponding to the candidate position in the mating and updating phase, and $F_{i}$ represents the objective function value corresponding to the current individual’s position. Using this approach, the algorithm can generate new solutions based on different mating distances, thereby further improving its local search capabilities and accuracy.

#### 2.2.4. Analysis of the Characteristics and Limitations of the OOA Search Mechanism

OOA establishes a search mechanism that integrates global exploration with local exploitation. Specifically, global search is conducted through the stages of potential prey search and natural enemy avoidance: The potential prey search phase enhances the population’s ability to cover the search space, while the predator evasion phase employs a water-jet propulsion mechanism to increase the search step size, thereby making it easier for the algorithm to escape local optima. Local development is executed by the prey attack and mating update phases: During the prey attack phase, individuals continuously converge toward dominant regions under the guidance of both individual and global optima; the mating and updating phase, conversely, enhances search precision through local recombination and a greedy selection strategy. In summary, through the synergistic interaction of various behaviors, OOA achieves a fundamental balance between exploration and development.

The original OOA exhibits several limitations:

1. Random initialization often results in uneven population distribution, which restricts coverage of the solution space during early search stages and reduces the likelihood of generating high-quality initial solutions.

2. Position updates are based mainly on fixed rules and lack a flexible, self-adaptive mechanism that responds to the population’s evolutionary state. As a result, the algorithm demonstrates limited ability to maintain a dynamic balance between exploration and exploitation.

3. As the population converges, diversity decreases, which in later stages diminishes the algorithm’s capacity for local fine-grained searches and ultimately reduces the accuracy of the final solution.

4. In complex multi-modal optimization problems, the algorithm does not include robust perturbation mechanisms or effective strategies for preserving diversity. Consequently, its ability to escape local optima is constrained.

In summary, the original OOA is constrained by suboptimal initialization, insufficient dynamic adjustment, diminished late-stage fine-tuning, and inadequate mechanisms for escaping local optima. These observations indicate potential directions for future enhancements to the OOA algorithm.

### 2.3. IOOA-SVM Parameter Optimization Framework

Training the classifier on the full training set for each candidate solution during parameter optimization incurs high computational costs. To address this issue, a lightweight fitness evaluation mechanism was introduced. Stratified sampling within each class of the training set was used to generate smaller training and validation subsets. The classification error rate, calculated using the candidate parameters on the internal validation set, is employed as the fitness function [30]:

(15) $$f\left(x\right)=\frac{1}{N_{v}}\sum_{i=1}^{N_{v}}\delta_{i}$$

Among them,

(16) $$\delta_{i}=\left\{\begin{array}{c} 1,\;\;\;\;\hat{y_{i}}\neq y_{i}\\ 0,\;\;\;\;\hat{y_{i}}=y_{i} \end{array}\right.$$

In the formula, $N_{v}$ represents the total number of internal validation samples, and $\hat{y_{i}}$ and $y_{i}$ denote the predicted label and true label of the i-th sample, respectively. When a sample’s predicted label matches its true label, the indicator function $\delta_{i}$ for that sample is 0; when a sample’s predicted label does not match its true label, the indicator function $\delta_{i}$ for that sample is 1. The better the classification performance of the current parameter combination, the smaller the classification error rate $f\left(x\right)$. Based on its ability to determine whether a parameter combination is superior, this mechanism effectively reduces the time cost of a single fitness evaluation.

The IOOA algorithm is incorporated into the support vector machine (SVM) hyperparameter optimization process. Each individual in the IOOA population encodes a candidate parameter set. These parameters are refined through iterative population updates, with the current optimal solution identified and retained based on fitness values. The parameter combination with the highest fitness value is selected as the optimal set for the optimization run after the iterations are complete. This optimal parameter set is subsequently applied to the SVM classifier, which is trained on the training dataset. The classification performance of the resulting SVM model is then evaluated using the test dataset. This approach establishes a parameter-optimization framework for IOOA-SVM in hyperspectral image classification, enabling automatic tuning of key parameters and improving SVM performance.

**Figure 1:**
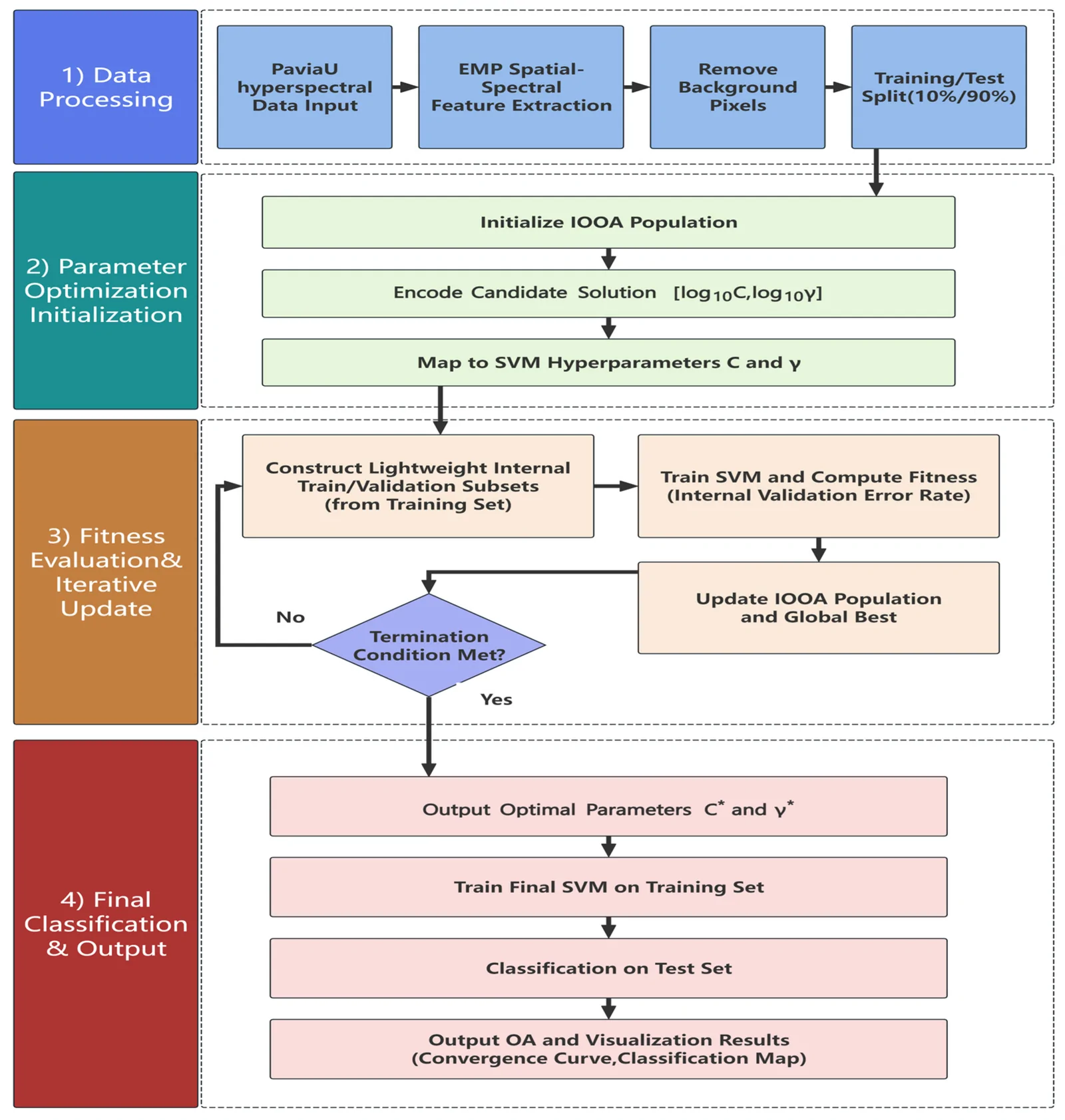
Flowchart of the IOOA-SVM hyperparameter optimization framework for hyperspectral image classification. * The superscript * denotes the optimized values of the corresponding parameters.

Figure 1 presents the flowchart of the hyperparameter optimization process for hyperspectral image classification, clearly depicting the proposed methodology.

## 3. Improved Octopus Optimization Algorithm (IOOA)

This paper proposes an Improved Octopus Optimization Algorithm (IOOA) to address the limitations of the original OOA in complex optimization problems through a problem-specific and mechanism-matching collaborative improvement framework. This algorithm centers on designing a collaborative improvement framework for the SVM hyperparameter optimization task aimed at hyperspectral image classification, addressing the shortcomings of the original algorithm while ensuring mechanism matching. Based on the search process of the original algorithm and its underlying flaws, it introduces four collaborative improvement strategies, forming a comprehensive improvement chain consisting of initialization enhancement, search balance adjustment, local refinement, and stagnation recovery. The specific improvements comprise an elite-based, opposition-based learning population initialization strategy; a multi-stage, nonlinear, adaptive parameter control mechanism; an elite-guided differential mutation local search strategy; and a stagnation-monitoring-based Lévy flight restart mechanism, combined with a stable boundary handling strategy. As shown in Table 1.

### 3.1. Elite-Based Opposition-Based Learning Population Initialization Strategy

The original OOA uses a random generation method to initialize the population during the initialization phase. Although this method is simple to implement, it tends to result in an uneven distribution of individuals within the population, which in turn leads to limited coverage of the solution space during the early stages of the search, thereby affecting the generation of high-quality initial solutions. Therefore, this paper introduces a population initialization strategy based on elite backpropagation during the initialization phase to address the aforementioned issues. The core idea is to generate a corresponding reverse population based on the original population, thereby enhancing population diversity by expanding the initial pool of candidate individuals. Subsequently, a fitness-based selection mechanism is employed to retain the superior individuals, thereby improving the overall quality of the original population.

Let the position of the i-th individual in the j-th dimension be $x_{i,j}$, and let the lower and upper bounds of the search interval be ${lb}_{j}$ and ${ub}_{j}$, respectively. Then its reverse individual can be expressed as

(17) $${\bar{x}}_{i,j}={lb}_{j}+{ub}_{j}-x_{i,j},i=1,2,\cdots ,j=1,2,\cdots ,D.$$

After generating the reverse population, it is merged with the original population to form a candidate set. The fitness of all candidate individuals is evaluated, and individuals are ranked in descending order of fitness. The highest-ranking individuals are selected as the initial population. This approach enables the algorithm to perform an initial screening within the expanded candidate space before iteration, thereby improving the quality of the initial population.

This strategy enhances the initialization process of the original OOA in two primary ways. First, introducing reverse individuals broadens the distribution range of the initial sample, increasing the population’s coverage and diversity within the search space. Second, the selection and retention mechanism ensures that individuals with higher fitness progress to subsequent iterations, providing the algorithm with a higher-quality initial search foundation. As a result, this strategy improves the algorithm’s global search capabilities and promotes early convergence.

**Figure 2:**
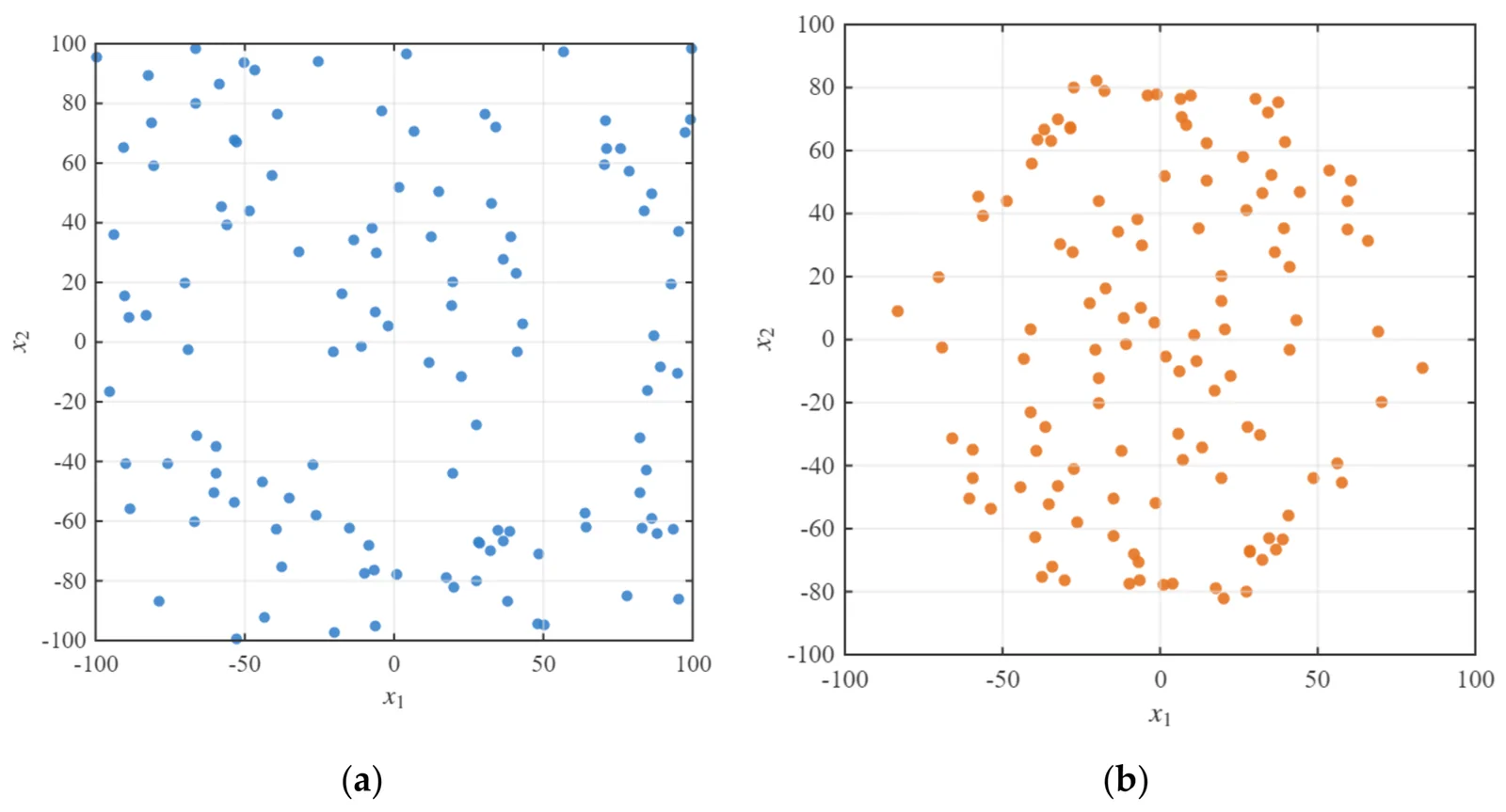
Comparison of 2D distributions: random initialization vs. initialization filtered via elite opposition-based learning. (**a**) Random initialization; (**b**) elite opposition-based selected initialization.

Figure 2 presents a two-dimensional comparison of the initial population distributions generated by the elite back-propagation initialization strategy and by random initialization.

Figure 2 illustrates that individuals produced by random initialization are widely distributed throughout the search space, which does not ensure a high-quality initial population. An elite-based opposition learning strategy is implemented during initialization to address this. Opposite individuals are first generated, and candidates are then selected according to their fitness values. This approach retains more competitive individuals, thereby enhancing the quality of the initial population and establishing a strong basis for subsequent global search and local optimization.

### 3.2. Multi-Stage Nonlinear Adaptive Parameter Control Mechanism

The original OOA regulates individual motion during the search process through the time-varying parameter a, the recoil acceleration al, and the fluid resistance term, thereby achieving dynamic control of search behavior to a certain extent. However, the method for adjusting its parameters still relies primarily on presets, with the damping coefficient wdamp set to a fixed value. As a result, the algorithm cannot effectively adjust the search intensity at different stages of the iteration, making it unable to achieve a more flexible balance between global search and local exploration. This paper proposes a multi-stage nonlinear adaptive parameter control mechanism, in which key control parameters are dynamically adjusted across different search stages to improve the algorithm’s adaptive search capability and balance exploration and exploitation.

Let the current iteration number be it, and the maximum number of iterations be MaxIt. Define the normalized time variable as

(18) $$t=\frac{it}{MaxIt}$$

The key control parameters in the IOOA are updated to

(19) $$a=2\left(1-t^{0.8}\right)$$

(20) $$al=5000t^{1-\sqrt{max\left(t,\varepsilon \right)}}$$

(21) wdamp=1.8−1.3t

(22) $$F\left(t\right)=max\left\{\frac{1}{2}wdamp{\left(1-t\right)}^{1-e^{-t}},0.02\right\}$$

Specifically, a is used to adjust the switching intensity during the search phase; al represents the acceleration term in the recoil motion; wdamp is the dynamic damping coefficient; $F\left(t\right)$ is the fluid resistance adjustment term in the velocity update; and ε is a very small positive constant used to enhance the stability of numerical calculations.

This mechanism enhances the search adaptability at different iteration stages through the evolution of nonlinear parameters compared to the original OOA. In the early stages of the iteration, a and $F\left(t\right)$ are set to relatively large values, which helps increase the population’s range of motion and search activity, thereby expanding the scope of exploration in the solution space; as the iteration progresses, a, wdamp, and $F\left(t\right)$ are gradually reduced, and the algorithm transitions from a broad-scale search to a refined search in the vicinity of the dominant region, thereby improving the stability and precision of local optimization. Consequently, the multi-stage nonlinear adaptive parameter control mechanism effectively addresses the relatively limited nature of the original OOA parameter tuning approach, thereby enhancing the algorithm’s ability to dynamically regulate the balance between global search and local optimization.

### 3.3. Elite-Guided Differential Mutation Local Search Strategy

The original OOA algorithm achieved local optimization by simulating octopus hunting and mating behaviors during the iterative process. However, the algorithm’s late-stage search continued to rely primarily on predefined behavioral rules as the population converged toward favorable regions. As a result, its capacity for fine-grained local optimization remained limited, leading to insufficient convergence accuracy. The present study introduces an elite-guided differential mutation local search strategy following the main search process to address this limitation. This approach combines elite individual guidance with the DE/current-to-pbest/1 mutation mechanism [31], allowing the algorithm to further exploit potential optimal regions in later search stages and enhance both fine-grained local optimization and convergence accuracy.

Let the population size be N, and the current individual be $x_{i}$. First, sort the population in descending order of fitness, and construct the pbest set and the elite set, whose sizes are defined as

(23) $$N_{p}=max\left\{2,round\left(0.2N\right)\right\},\;\;\;\;\;N_{e}=max\left\{2,round\left(0.15N\right)\right\}$$

Next, a pbest individual $x_{pbest}$ is randomly selected from the previous $N_{p}$ individuals, and two individuals $x_{r_{1}}$ and $x_{r_{2}}$, different from the current individual are randomly selected from the population. Based on this, a mutation vector is generated using the current-to-pbest/1 mutation method.

(24) $$v_{i}=x_{i}+F_{de}\left(x_{pbest}-x_{i}\right)+F_{de}\left(x_{r_{1}}-x_{r_{2}}\right)$$

Here, $F_{de}$ represents the differential variation factor. A probabilistic elite attraction term is incorporated into the update process to strengthen the guiding effect of dominant information, namely

(25) $$v_{i}=v_{i}+\lambda \left(x_{e}-x_{i}\right)$$

Here, $x_{e}$ is a guide individual randomly selected from the original $N_{e}$ elite individuals, and λ=0.1×rand is the random guide coefficient.

The experimental individuals $u_{i}$ are generated using a binomial crossover after completing the mutation operation:

(26) $$u_{i,d}=\left\{\begin{array}{c} v_{i,d},\;\;\;\;rand\leq CR\;or\;d=j_{rand},\\ x_{i,d},\;\;otherwise, \end{array}\;\;\;\;d=1,2,\cdots ,D.\right.$$

Here, CR represents the crossover probability, and $j_{rand}$ is a randomly selected dimension index, used to ensure that the trial individual inherits information from the mutation vector in at least one dimension. Next, the trial individual is subjected to boundary constraints and fitness evaluation; if the fitness of $u_{i}$ is better than that of the current individual, $u_{i}$ replaces $x_{i}$, and both the individual optimum and the global optimum are updated accordingly.

In addition, the differential mutation factor and crossover probability are adaptively defined to regulate the local search intensity during the iterative process, namely

(27) $$F_{de}=clip\left(F_{{de}_{0}}+0.2randn,0.4,0.9\right)$$

(28) $$CR=clip\left({CR}_{0}-0.4t+0.1randn,0.2,0.95\right)$$

Here, $F_{{de}_{0}}$ and ${CR}_{0}$ denote the initial differential mutation factor and the initial crossover probability, respectively, while $clip\left(\cdot \right)$ represents the interval clipping operation.

The pbest-guided update directs individuals toward favorable regions, while the differential term maintains sufficient search perturbation and helps prevent premature population contraction. Moreover, the elite attraction term strengthens the algorithm’s ability to explore neighborhoods around high-quality solutions. At the same time, the two-way crossover and greedy selection mechanisms help preserve effective improvement directions and enhance the stability of local search [32]. Therefore, the elite-guided differential variation local search strategy effectively addresses the shortfall in the original OOA’s ability to perform fine-tuning in the later stages, thereby improving the algorithm’s convergence accuracy.

### 3.4. A Lévy Flight Restart Mechanism Based on Stagnation Monitoring and a Stable Boundary Handling Strategy

The iterative processes often lead individuals to converge toward favorable regions in complex multi-peaked optimization problems. However, some individuals repeatedly become trapped in local optima, reducing population diversity and potentially leading to evolutionary stagnation. Additionally, when updated solutions exceed search boundaries, unstable out-of-bounds handling mechanisms can compromise search quality and reduce algorithmic robustness. A stall-detection-based Lévy flight restart mechanism and a stable boundary-handling strategy are proposed to address these challenges. When an individual’s stagnation reaches a predefined threshold, a Lévy flight perturbation is applied. The elite information reconstruction is used to enhance population diversity and increase the ability to escape local optima if this perturbation does not result in improvement. A unified and stable boundary-handling strategy is implemented to improve overall search stability for individuals that exceed search boundaries during optimization.

Let $S_{i}$ be the stagnation count of the i-th individual; then the stagnation threshold is defined as

(29) $$S_{max}=max\left\{8,round\left(0.08MaxIt\right)\right\}$$

Here, MaxIt denotes the maximum number of iterations. When $S_{i}\geq S_{max}$, the individual is considered to have entered a stagnant state, and a restart operation must be triggered. This mechanism is applied only to a subset of inferior individuals in the population to avoid imposing strong perturbations on the entire population simultaneously. Specifically, after the population is ranked by fitness, approximately the bottom 30% of individuals are selected to form the restart set, and only those individuals that satisfy the stagnation condition are processed.

This paper first applies perturbations using Lévy flight for individuals that trigger a restart. Let the current global optimal individual be $x_{g}$. The update for the i-th individual in the j-th dimension can be written as

(30) $$x_{i,j}^{\prime }=x_{i,j}+L_{j}\alpha \left(t\right)\left(x_{i,j}-x_{g,j}\right)+0.1\xi_{j}\left({ub}_{j}-{lb}_{j}\right)$$

Here, $L_{j}$ denotes the random step component generated by the Lévy flight in the j-th dimension, $\xi_{j}$ is a standard normal random variable, ${ub}_{j}$ and ${lb}_{j}$ represent the upper and lower bounds of the variable in the j-th dimension, respectively, and $\alpha \left(t\right)$ is a step-size scale that varies dynamically with the iteration, defined as

(31) $$\alpha \left(t\right)=\alpha_{0}\left[1+4\left(1-t\right)\right],\;\;\;\;\;\;\alpha_{0}=0.01\cdot mean\left(ub-lb\right)$$

In this context, t=it/MaxIt represents the normalized iteration progress. This design allows the algorithm to operate with a wide perturbation range in the early stages of the iteration and gradually reduces the perturbation magnitude in the later stages, thereby balancing the ability to escape local optima with the stability of the local search.

Apply boundary constraints and fitness evaluation to the perturbed individuals; if their fitness is superior to that of the original individuals, the replacement is completed immediately, and the stagnation count is reset. If the Lévy flight perturbation does not yield a better solution, candidate solutions are regenerated using an elite-average-based reconstruction method, whose j-dimensional form is

(32) $$x_{i,j}^{\prime \prime }={\bar{x}}_{e,j}+0.2\eta_{j}\left({ub}_{j}-{lb}_{j}\right)$$

Here, ${\bar{x}}_{e,j}$ denotes the mean position of the set of elite individuals in the j-th dimension, and $\eta_{j}$ is a standard normal random variable. If the reconstructed individual is superior to the original, it replaces the original; regardless of whether the reconstruction result is accepted, the individual’s stagnation count is reset to zero, allowing it to participate in subsequent evolution.

The algorithm utilizes a stable boundary-handling strategy to correct individuals that exceed the feasible region, thereby preserving the quality and stability of subsequent searches. Unlike conventional methods such as positional truncation or velocity mirroring, this strategy ensures solution feasibility and minimizes oscillations near boundaries, which enhances the algorithm’s stability and robustness in complex search spaces [33]. Incorporating this strategy allows the algorithm to selectively restart low-quality individuals during stagnation, thereby maintaining a more stable boundary search throughout the optimization process. The alternating long- and short-step random walk characteristic of the Lévy walk increases the probability that individuals escape local optima and re-explore high-quality regions of the solution space. The elite-average reconstruction utilizes superior information, while the stable boundary-handling strategy further refines the feasible region of each individual’s solution space. Collectively, these mechanisms enhance the effectiveness of the search process, improve the algorithm’s ability to escape local optima, restore population diversity, and increase overall robustness.

### 3.5. Analysis of the Synergistic Mechanisms of Various Improvement Strategies

The aforementioned improvement strategies act on different stages of population evolution and together form a collaborative optimization mechanism.

**Figure 3:**
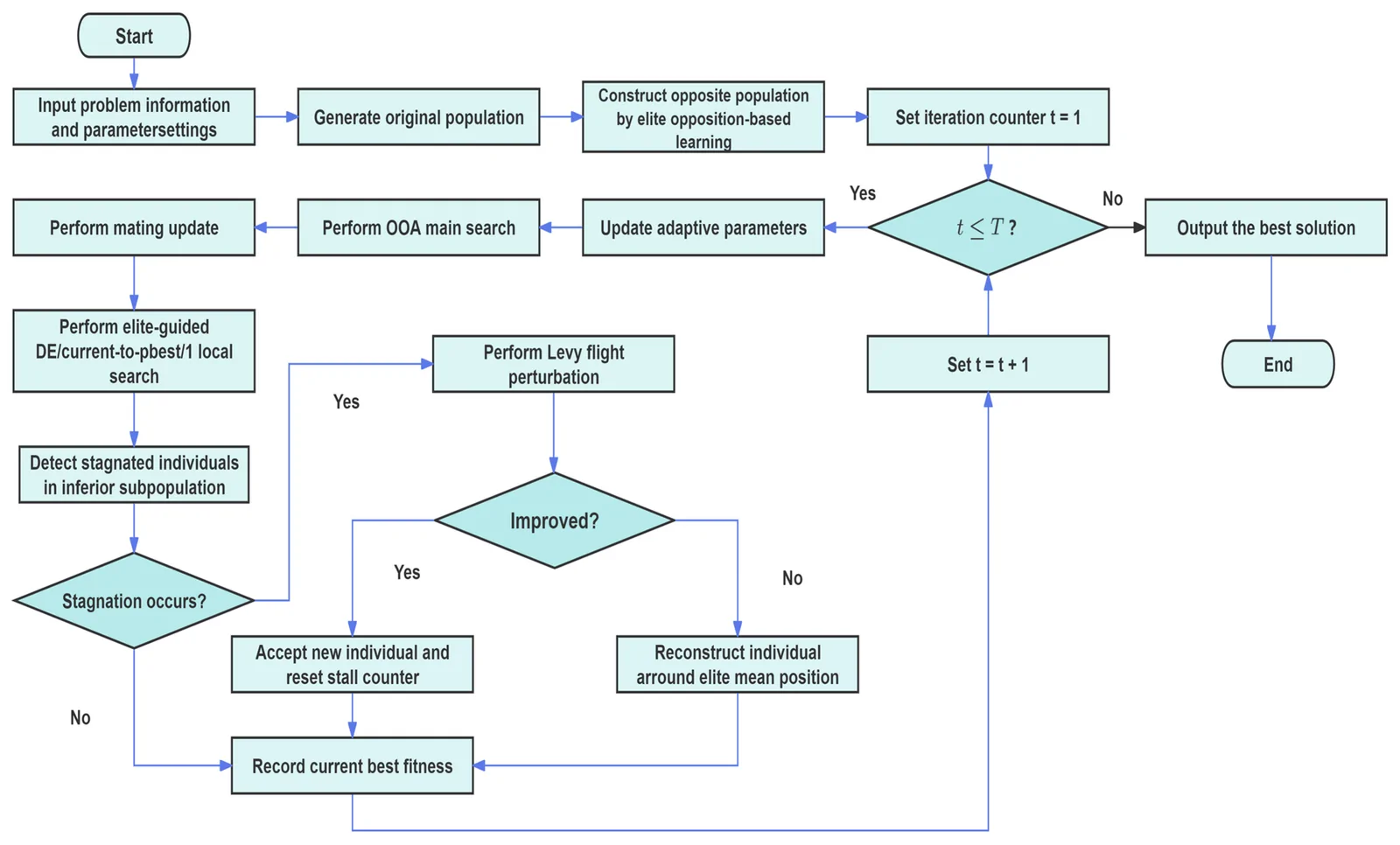
Flowchart of the Improved Octopus Optimization Algorithm (IOOA).

The elite backpropagation initialization strategy primarily improves the quality of the population in the early stages of the search; the adaptive parameter control mechanism primarily improves the exploration-exploitation balance during the middle phase; the elite-guided differential variation local search strategy primarily enhances late-stage local exploration capabilities and convergence accuracy; the Lévy flight restart mechanism is used to restore population activity in the middle and late stages and enhance the ability to escape local optima. Through the synergistic effects of the aforementioned strategies, IOOA has established an optimization framework comprising “initial enhancement—mid-term dynamic regulation—late-stage fine-tuning—stagnation recovery assurance,” thereby achieving a comprehensive improvement in global search capabilities, local development capabilities, and overall stability. The flowchart of the IOOA algorithm is shown in Figure 3.

### 3.6. Time Complexity Analysis

Let the population size be N, the problem dimension be D, the maximum number of iterations be T, and the computational complexity of a single fitness calculation be denoted as $O\left(f\right)$. The computational overhead of the original OOA primarily stems from population initialization, position updates, mating updates, and fitness evaluation; its overall time complexity can be expressed as

(33) $$O\left(T\left(N\left(D+f\right)+NlogN\right)\right)$$

For IOOA, elite-guided backpropagation initialization adds candidate evaluation and ranking; elite-guided differential mutation local search adds mutation, crossover, and fitness calculation; the Lévy flight restart mechanism perturbs and reconstructs some stagnant individuals; and adaptive parameter updating incurs only a constant-time overhead. Therefore, the overall time complexity of IOOA is the same as that of the original OOA. 

Therefore, although the improved strategy introduces additional computational operations, it does not alter the algorithm’s main complexity order; IOOA and the original OOA remain in the same order of magnitude in terms of time complexity.

## 4. Comparative Experimental Analysis of IOOA Algorithms

### 4.1. Experimental Environment and Algorithm Parameter Settings

This paper conducts comparative experiments on the CEC2017 benchmark function set to validate the optimization performance of IOOA. All experiments were conducted on the Windows 11 operating system using MATLAB R2025b, with a hardware configuration consisting of an Intel Core i5-10200H CPU @ 2.40 GHz and 16 GB of RAM. All algorithms were tested under a uniform experimental setup to ensure the fairness and comparability of the results: dimensionality Dim = 30, population size N = 30, maximum number of iterations T= 500, and each function was run independently 30 times. The termination condition for all algorithms was reaching the maximum number of iterations.

This paper selects the original Octopus Optimization Algorithm (OOA), Particle Swarm Optimization (PSO), Grey Wolf Optimization (GWO), Salp Swarm Algorithm (SSA), Arithmetic Optimization Algorithm (AOA), Sand Cat Swarm Optimization (SCSO), Differential Evolution (DE) and Linear Population Size Reduction Success-History Based Adaptive Differential Evolution (L-SHADE) as comparative algorithms; the specific parameters for each algorithm are presented in Table 2.

In particular, IOOA retains the basic parameter settings of OOA while introducing improved parameters such as $pBestRate=0.2,\;\;elite\_keep\_rate=0.15,\;\;TF_{DE}^{0}=0.7$ and ${CR}_{0}=0.9$. This paper employs a PSO with linearly decreasing inertial weights, where the inertial weight ω decreases linearly from 0.9 to 0.2. The formula for this decrease is $\omega =\omega_{max}-t\cdot \frac{\omega_{max}-\omega_{min}}{maxIter}$.

### 4.2. Introduction to the CEC2017 Test Function Set

The performance of the algorithm is evaluated using the CEC2017 benchmark function set, which is a widely recognized standard platform for testing swarm intelligence optimization methods. This function set provides a representative, standardized, and comparable basis for analyzing the performance of different algorithms. The CEC2017 function set includes complex features such as multiple peaks, variable couplings, translations, rotations, and mixed combinations. These characteristics enable a comprehensive evaluation of an optimization algorithm’s convergence accuracy, global search capability, local exploration capability, and robustness. Consequently, the CEC2017 function set is suitable for validating the overall optimization performance of the proposed algorithm under complex search conditions. The F2 function demonstrates instability and is therefore excluded from the analysis as noted in the official CEC2017 technical report. The remaining 29 functions are selected as test subjects. The functions used include two unimodal functions (F1 and F3), seven simple multimodal functions (F4–F10), 10 hybrid functions (F11–F20), and 10 composite functions (F21–F30); see Table 3 for details.

In particular, the hybrid functions F11–F20 divide the decision variable into multiple subcomponents, each of which employs a different basis function, and are constructed by introducing operations such as variable substitution, translation, and rotation. This class of functions integrates different types of local search landscapes and complex variable couplings, providing an effective evaluation of the algorithm’s ability to balance global exploration and local exploitation. In CEC2017, the theoretical optimal value of the function $F_{i}^{*}$ is set to $F_{i}^{*}=100i$; therefore, the theoretical optimal values of F11–F20 are 1100–2000, respectively.

### 4.3. Set Comparative Analysis of Algorithm Performance

In this paper, each algorithm was run 30 times independently under the same experimental conditions. Table 4 shows the mean, standard deviation, best result, and worst result for each algorithm across the 29 test functions on the CEC2017 test set. The mean represents the average optimization accuracy, the standard deviation indicates the algorithm’s stability, and the best and worst values represent the best and worst performance, respectively, across multiple independent runs. For all functions, the lower the fitness value, the closer the result is to the theoretical optimal value, indicating better optimization performance. 

As shown in Table 4, IOOA achieved the vast majority of the optimal results in experiments on all 29 test functions. The IOOA average achieved the best results on 27 of the 29 test functions; it ranked second on F6, slightly behind DE, and second on F27, slightly behind L-SHADE. At the same time, IOOA outperformed the original OOA in terms of average performance across all 29 functions, indicating that the proposed improvement strategy can consistently enhance OOA’s overall optimization capability. Notably, IOOA maintained a consistent advantage on both Hybrid and Composition functions. IOOA achieved significant performance improvements on F12, F13, F15–F20, F26, F29, and F30, compared with the original OOA algorithm, supporting the effectiveness of the comprehensive improvement strategy proposed in this study.

IOOA also achieved better results in terms of standard deviation across 17 functions, ranking second on nine of them. Although DE and L-SHADE exhibit smaller standard deviations for individual functions such as F6, F25, F27, and F28, IOOA generally achieves better mean, maximum, and minimum values while maintaining a low standard deviation. This indicates that its advantages are not merely coincidental but reflect consistent results across multiple independent experiments. In particular, for complex functions such as F12–F20, as well as F29 and F30, the IOOA demonstrated outstanding performance both on average and in worst-case scenarios, indicating strong stability, lower variability in results, and better performance.

Overall, IOOA achieved the best results on average for 27 of the 29 test functions, outperforming the original OOA on all functions and demonstrating superior performance in most results regarding standard deviation, best values, and worst values. Although other algorithms yield better results for certain metrics in specific functions, this does not affect IOOA’s leading position in overall optimization performance. Taken together, the results show that IOOA exhibits high convergence accuracy, strong global search capabilities, and good operational stability, and demonstrates the algorithm’s robustness and reliability in complex optimization problems.

**Figure 4:**
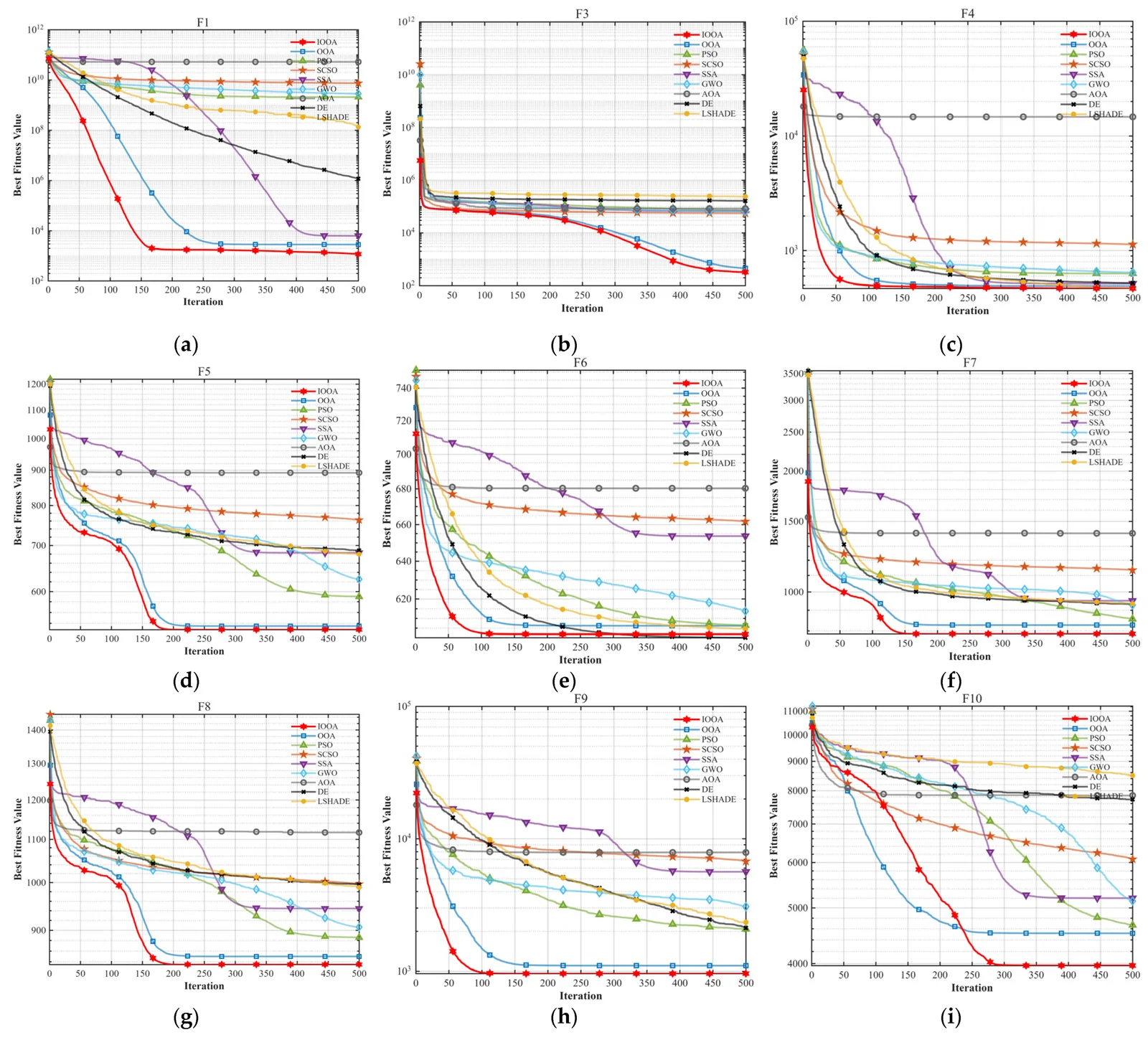

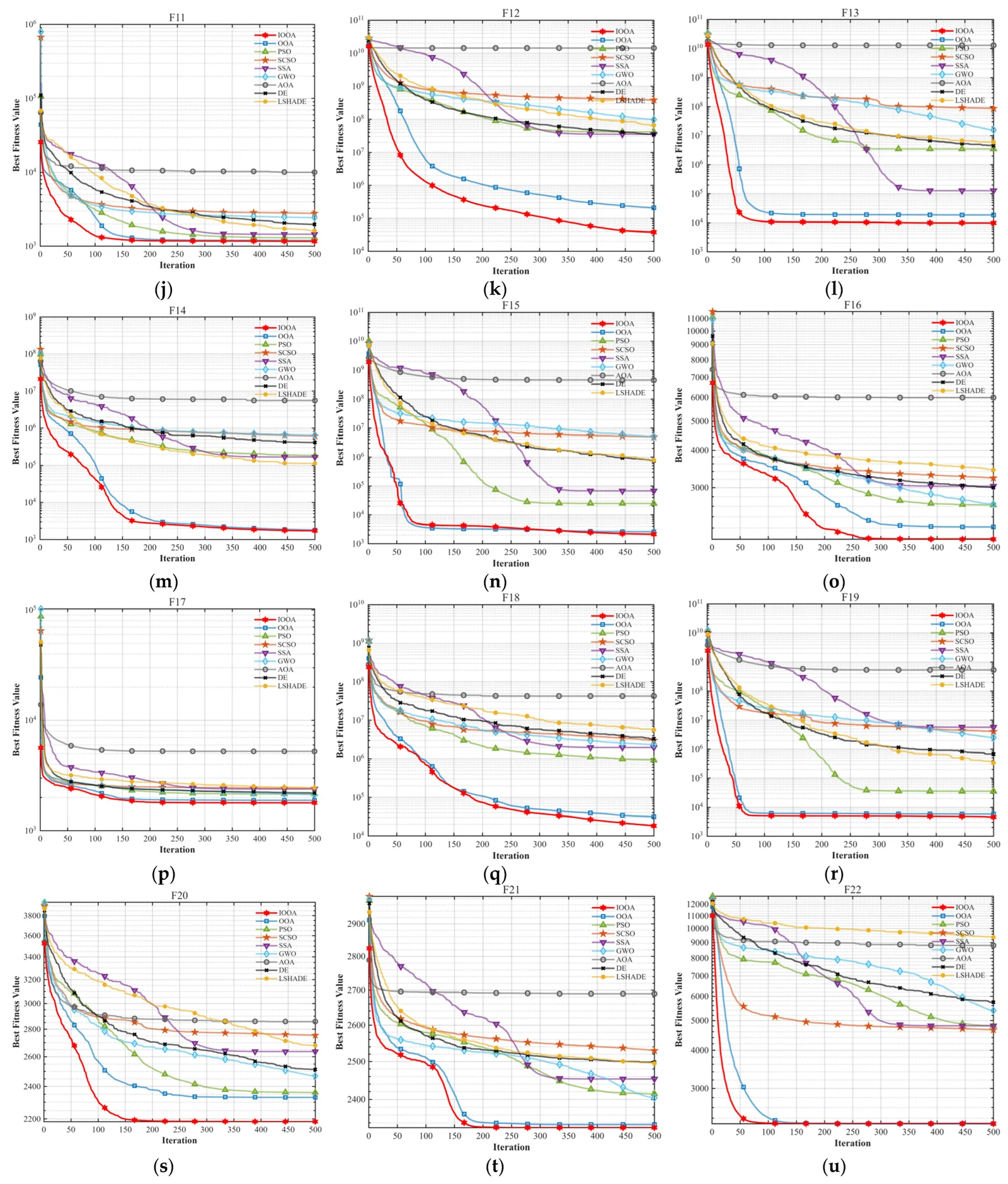

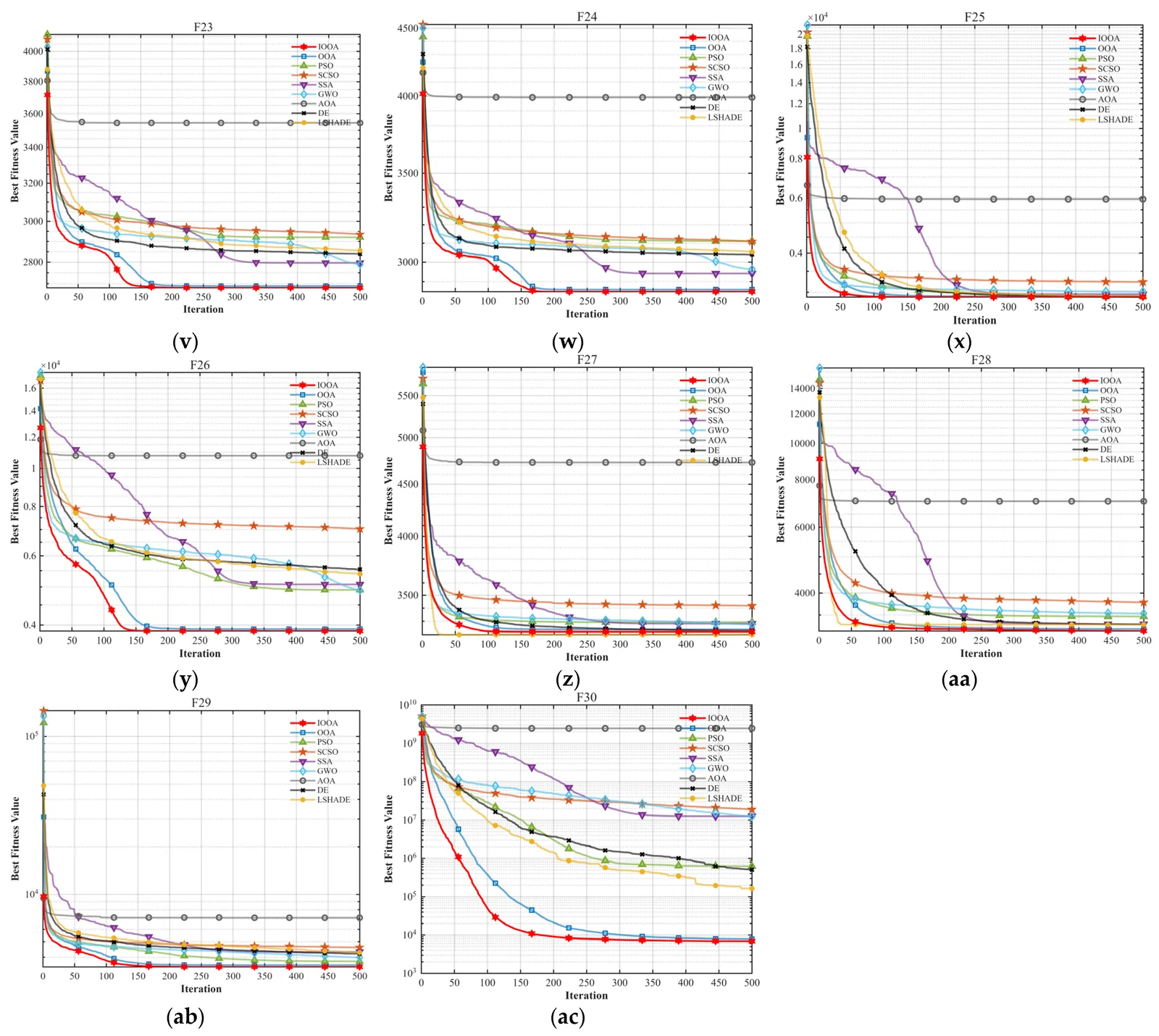
Convergence curves of various algorithms on the CEC2017 function: (**a**) The F1 convergence curve; (**b**) the F3 convergence curve; (**c**) the F4 convergence curve; (**d**) the F5 convergence curve; (**e**) the F6 convergence curve; (**f**) the F7 convergence curve; (**g**) the F8 convergence curve; (**h**) the F9 convergence curve; (**i**) the F10 convergence curve; (**j**) the F11 convergence curve; (**k**) the F12 convergence curve; (**l**) the F13 convergence curve; (**m**) the F14 convergence curve; (**n**) the F15 convergence curve; (**o**) the F16 convergence curve; (**p**) the F17 convergence curve; (**q**) the F18 convergence curve; (**r**) the F19 convergence curve; (**s**) the F20 convergence curve; (**t**) the F21 convergence curve; (**u**) the F22 convergence curve; (**v**) the F23 convergence curve; (**w**) the F24 convergence curve; (**x**) the F25 convergence curve; (**y**) the F26 convergence curve; (**z**) the F27 convergence curve; (**aa**) the F28 convergence curve; (**ab**) the F29 convergence curve; (**ac**) the F30 convergence curve.

Figure 4 illustrates that IOOA achieves both rapid and superior final convergence across the majority of test functions. Specifically, for the F1, F3–F9, F11–F26, F29, and F30 datasets, IOOA demonstrates a swift reduction in fitness values during the initial iterations. This early decrease allows IOOA to reach the dominant solution region more quickly, highlighting its effective initial search performance and operational efficiency.

The IOOA sustains a consistent decrease in fitness values throughout the middle and late optimization stages for complex functions, including F1, F3, F5, F7, F8, F10, F11, F12, F14–F21, F23, F24, F26, F29, and F30. In contrast, most comparison algorithms reach a plateau after a certain number of iterations, exhibiting limited further improvement and varying levels of stagnation. The IOOA converges more rapidly in the early stages and achieves more comprehensive optimization in the later stages compared with the original OOA, resulting in higher-quality final solutions. Additionally, IOOA convergence curves are generally smoother and exhibit less fluctuation across most functions, suggesting improved solution accuracy, enhanced search stability, and greater resistance to premature convergence.

In summary, the proposed improvement strategies significantly enhance the algorithm’s global search efficiency and fine-tuning capability during later stages. Consequently, IOOA exhibits rapid convergence, high convergence accuracy, and robust stability when solving complex optimization problems.

### 4.4. Stability and Robustness Analysis

**Figure 5:**
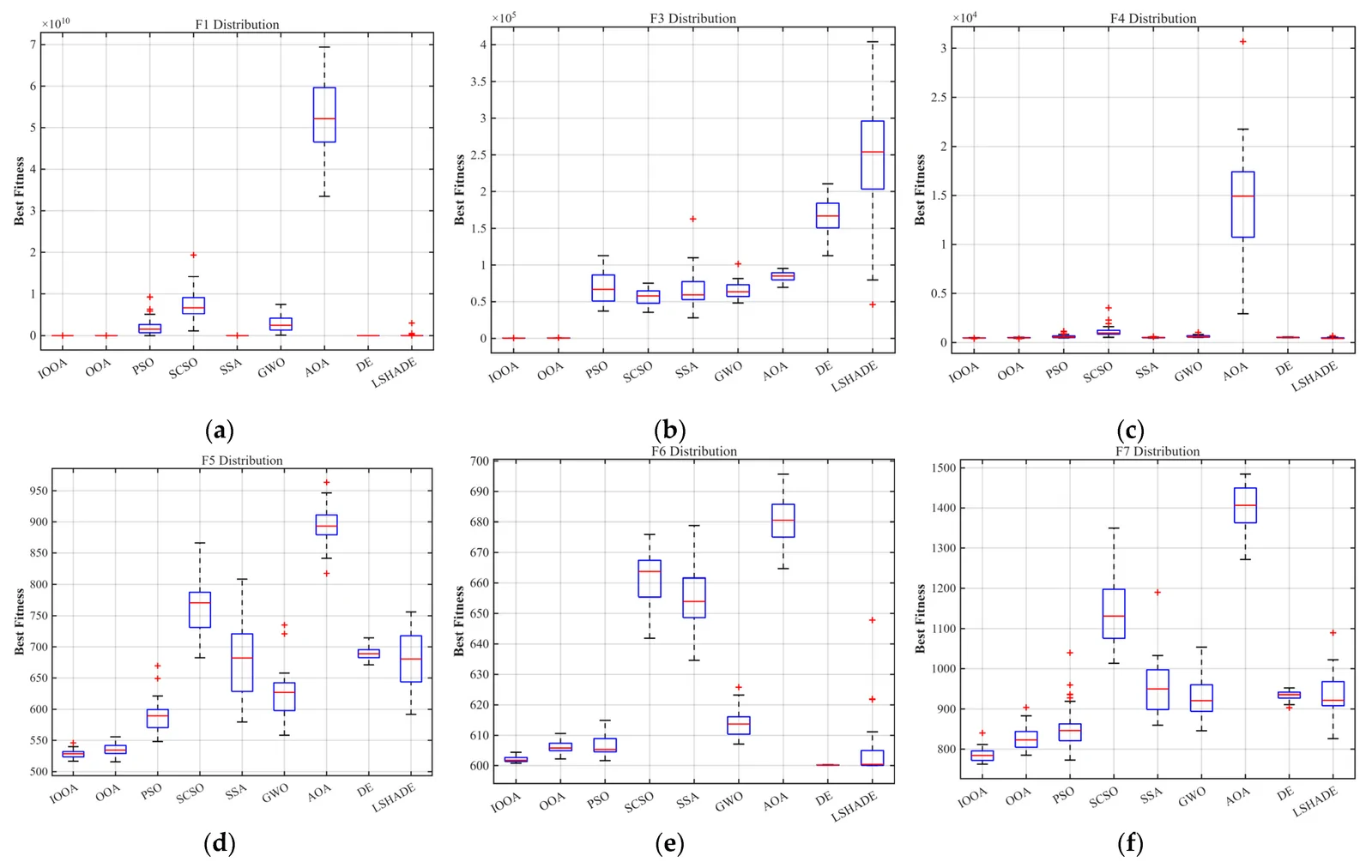

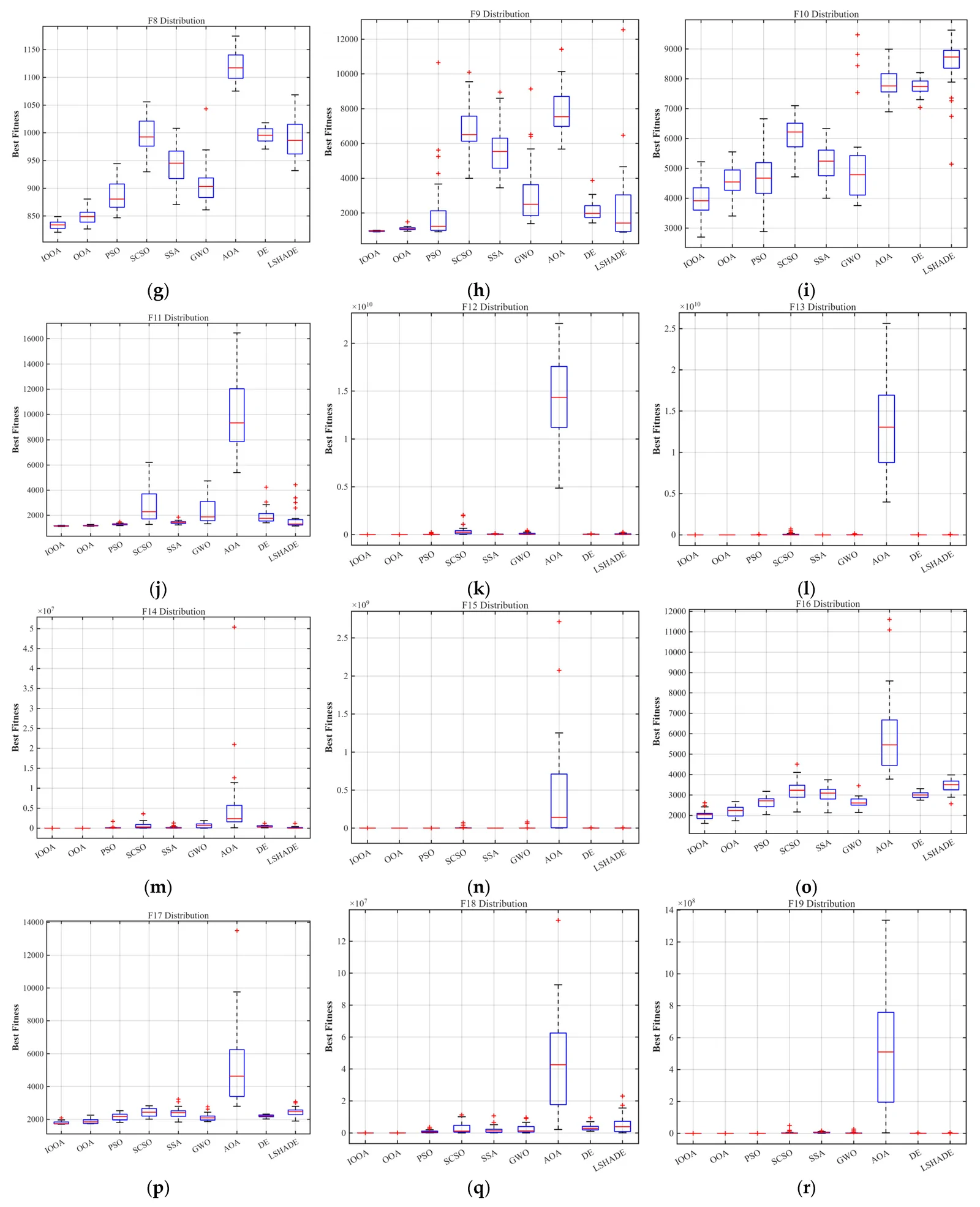

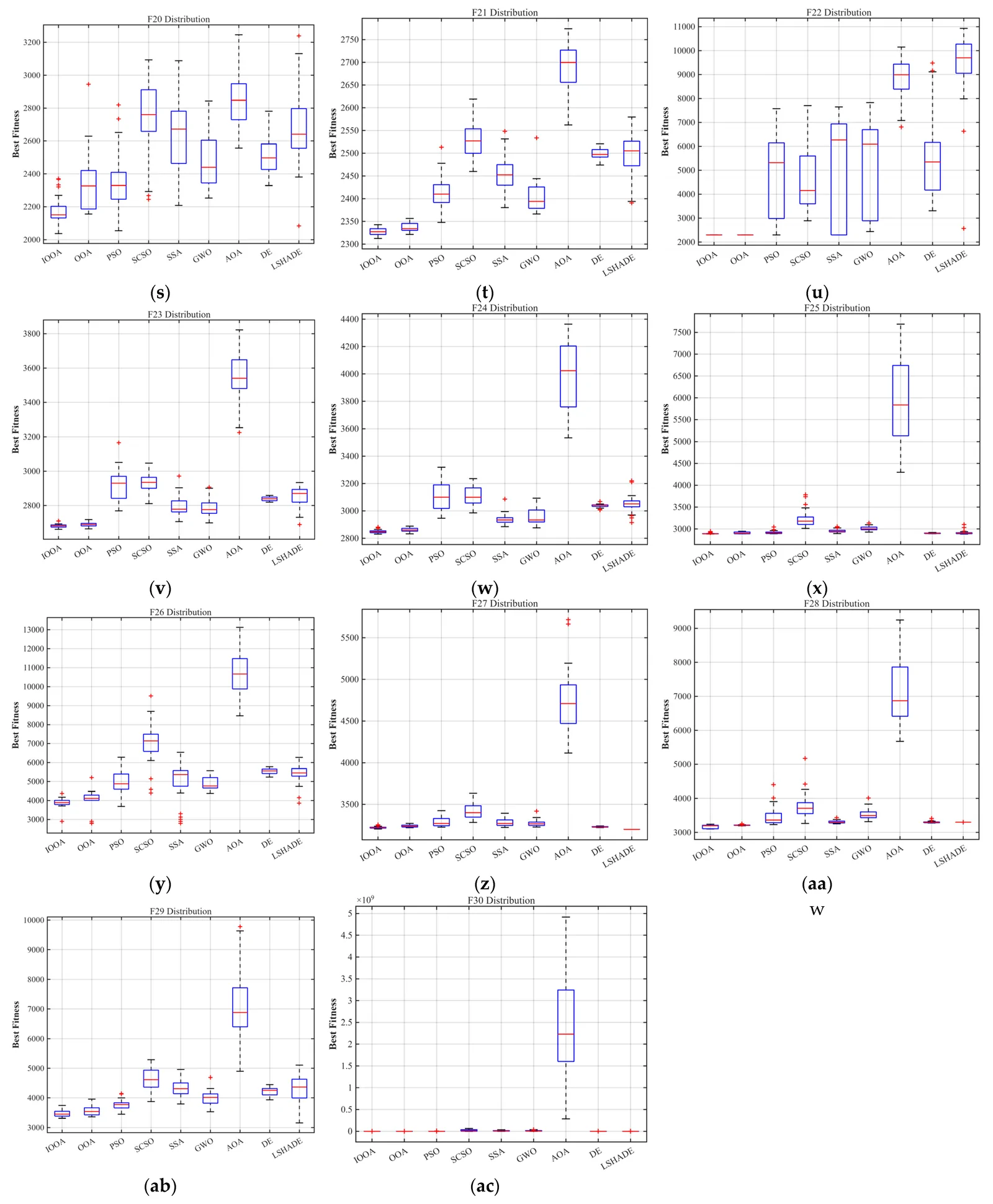
Box plots of various algorithms on the CEC2017 function: (**a**) The F1 box plot; (**b**) the F3 box plot; (**c**) the F4 box plot; (**d**) the F5 box plot; (**e**) the F6 box plot; (**f**) the F7 box plot; (**g**) the F8 box plot; (**h**) the F9 box plot; (**i**) the F10 box plot; (**j**) the F11 box plot; (**k**) the F12 cbox plot; (**l**) the F13 box plot; (**m**) the F14 box plot; (**n**) the F15 box plot; (**o**) the F16 box plot; (**p**) the F17 box plot; (**q**) the F18 box plot; (**r**) the F19 box plot; (**s**) the F20 box plot; (**t**) the F21 box plot; (**u**) the F22 box plot; (**v**) the F23 box plot; (**w**) the F24 box plot; (**x**) the F25 box plot; (**y**) the F26 box plot; (**z**) the F27 box plot; (**aa**) the F28 box plot; (**ab**) the F29 box plot; (**ac**) the F30 box plot.

Figure 5 shows that the box-and-whisker plots for IOOA are generally more compact across most test functions. Among the 29 test functions, IOOA displays a shorter box width and median values that remain at or near the lowest levels compared to other algorithms. The narrower range between the upper and lower quartiles indicates that IOOA achieves superior optimal values across multiple independent runs and maintains lower variability, suggesting greater stability relative to the other algorithms. For functions F1, F3–F11, F14, F16–F18 and F20–F29, IOOA’s interquartile range is markedly smaller than that of most comparison algorithms. Furthermore, most of these functions show no significant outliers for IOOA. In contrast, the five comparison algorithms—PSO, SCSO, SSA, GWO, AOA, DE and L-SHADE—exhibit longer boxes, more outliers, and greater overall volatility, which reflects lower stability. Compared to the original OOA algorithm, IOOA reduces the median optimal value while maintaining a narrow fluctuation range. These results demonstrate that the proposed comprehensive improvement strategy enhances both convergence accuracy and operational robustness.

In summary, the IOOA box plot is shorter, contains fewer outliers, and displays a more concentrated distribution of results. These findings indicate that the proposed improvement strategy effectively enhances the algorithm’s stability, robustness, and consistency.

### 4.5. Statistical Significance Test

The Wilcoxon signed-rank test was conducted based on the results of 30 independent runs to evaluate the statistical significance of the performance differences between IOOA and the comparison algorithms. When the dimension is 30, the test results are denoted by *p*. If *p* < 5%, a “+” indicates a statistically significant difference between the two algorithms; if *p* > 5%, a “−“ indicates that the difference between the two algorithms is not statistically significant. A NaN value indicates that the two algorithms perform equally well. Table 5 presents the *p*-values for IOOA compared to other algorithms.

As shown in Table 5, at a significance level of 0.05, the results of the Wilcoxon signed-rank test comparing IOOA with each of the comparison algorithms are mostly far less than 0.05, indicating that IOOA’s performance advantages are statistically significant. Compared to OOA, PSO, GWO, SSA, AOA, SCSO, and DE, IOOA achieved a result of 29/0/0 for +/NaN/−, indicating that IOOA outperformed these algorithms significantly on all 29 test functions. Compared to L-SHADE, OOA achieved a result of 28/0/1 for +/NaN/−, indicating that IOOA has a significant advantage over L-SHADE on 28 functions and is only slightly inferior to L-SHADE on one function. This phenomenon also aligns with the No-Free-Lunch Theorem, which states that no algorithm can consistently achieve the absolute optimal solution for all optimization problems.

Overall, the results of the Wilcoxon test confirm the statistical reliability of the performance improvement achieved by IOOA. Compared to the comparison algorithm, IOOA demonstrates a significant advantage on the vast majority of test functions, indicating that its improved performance is not due to random error but is statistically significant. Based on the results discussed above, it can be seen that IOOA demonstrates good overall performance in terms of solution accuracy, operational stability, and robustness.

### 4.6. Ablation Experiment

Ablation variants OOA1, OOA2, OOA3, and OOA4 were developed within the unified framework of the original OOA to assess the individual and combined contributions of each improvement strategy. Specifically, these variants incorporate the elite opposition-based initialization strategy, a multi-stage nonlinear adaptive parameter control mechanism, an elite-guided differential mutation strategy, and a stagnation-monitored Lévy flight restart mechanism with stable boundary handling, respectively. The complete IOOA integrates all four strategies. Twelve representative functions from CEC2017 were selected for comparison, and a comprehensive analysis of the performance of each variant was conducted using mean values, standard deviations, optimal values, and convergence curves.

Table 6 demonstrates that IOOA achieved the highest average performance across all 12 test functions and exhibited the lowest standard deviation on 11 of them. These results indicate that the proposed comprehensive improvements enhanced both solution accuracy and the algorithm’s stability and robustness. For representative functions, the average values of IOOA for F1, F18, F20, and F29 decreased from OOA’s $1.6832\times 10^{3}$, $2.7623\times 10^{4}$, $2.3284\times 10^{3}$, and $3.5920\times 10^{3}$ to $1.1867\times 10^{3}$, $1.9815\times 10^{4}$, $2.1846\times 10^{3}$, and $3.4434\times 10^{3}$, respectively, demonstrating a relatively stable overall advantage.

**Figure 6:**
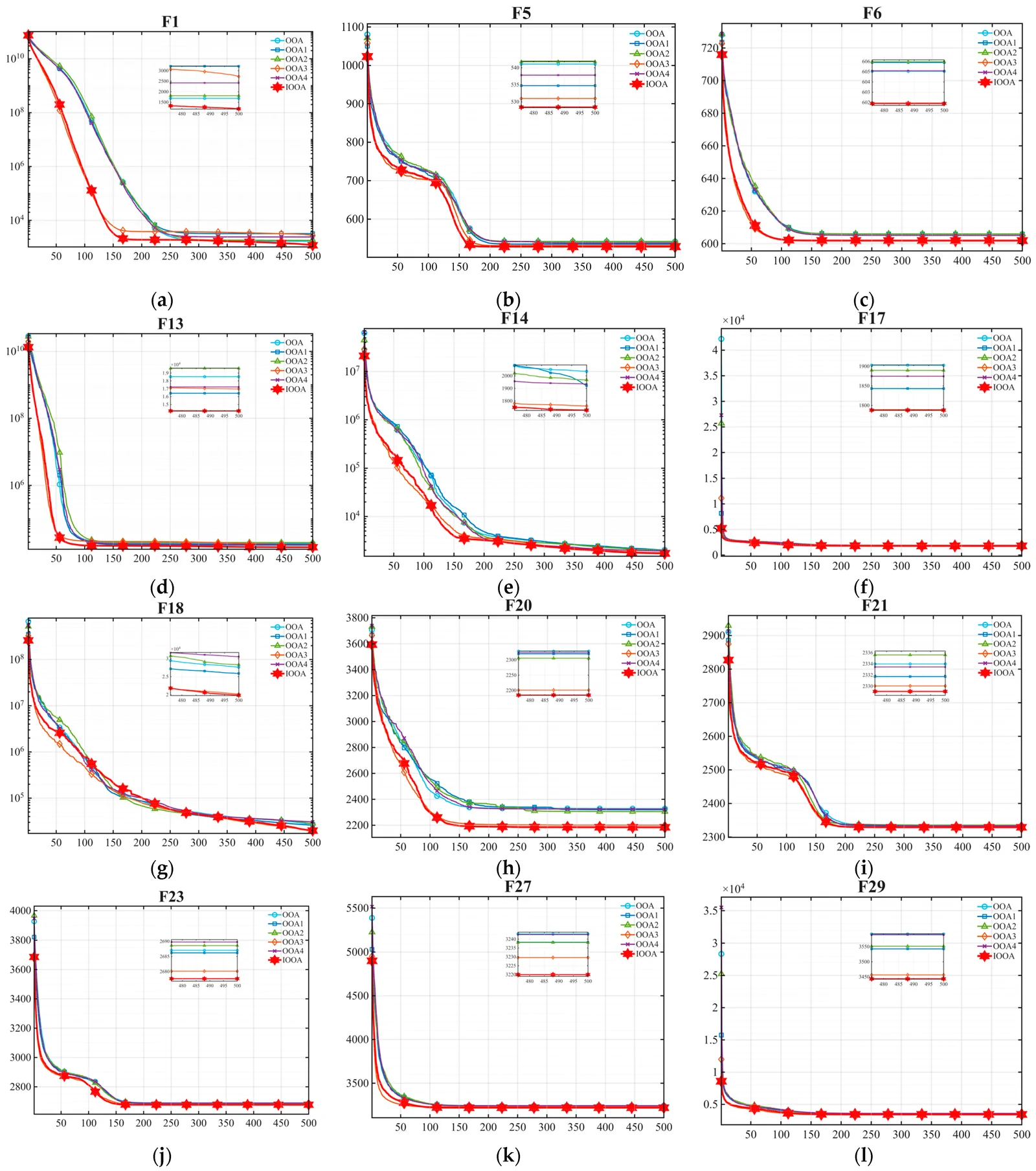
Convergence Curve of the Ablation Experiment: (**a**) F1 Ablation Convergence Curve; (**b**) F5 Ablation Convergence Curve; (**c**) F6 Ablation Convergence Curve; (**d**) F13 Ablation Convergence Curve; (**e**) F14 Ablation Convergence Curve; (**f**) F17 Ablation Convergence Curve; (**g**) F18 Ablation Convergence Curve; (**h**) F20 Ablation Convergence Curve; (**i**) F21 Ablation Convergence Curve; (**j**) F23 Ablation Convergence Curve; (**k**) F27 Ablation Convergence Curve; (**l**) F29 Ablation Convergence Curve.

Analysis of the single-strategy variants indicates that OOA3 achieved the greatest improvement, delivering optimal average performance across 10 out of 12 functions. This result suggests that the elite-guided differential variation strategy in OOA3 is the primary mechanism responsible for enhancing late-stage local exploration and search accuracy, serving as the core component of the integrated strategy proposed in this study. Although OOA4 did not yield the best average and variance across all functions, it achieved optimal results for functions F13 and F14. This finding demonstrates that the Lévy flight restart mechanism, based on stopping criteria, and the stable boundary handling strategy effectively improve the algorithm’s ability to escape local optima. The initialization strategy based on elite backpropagation (OOA1) and the multi-stage adaptive parameter control (OOA2) did not consistently outperform the original OOA on specific functions, indicating that the benefits of each individual strategy are not uniform across different function types. However, when these strategies are integrated into IOOA, the algorithm demonstrates superior overall convergence accuracy and result stability. These results suggest that the proposed improvement mechanisms function as complementary components at each stage, collectively enhancing the algorithm’s overall performance. As illustrated in Figure 6, the convergence curves indicate that IOOA converges faster and achieves lower convergence error across most functions, reflecting a more effective balance between global search and local exploration.

## 5. Applications of IOOA in Hyperspectral Image Classification

### 5.1. Problem Description

Hyperspectral images differ from conventional images by providing a greater number of spectral bands, increased dimensionality, and more complex spectral variations between classes. Although these characteristics offer substantial information for land cover classification, they also introduce significant complexity to classification modeling [34]. Support vector machines (SVMs) are frequently employed in hyperspectral image classification due to their robust generalization capabilities and effectiveness with small datasets. However, the classification accuracy of SVMs is highly sensitive to parameter tuning; improper parameter selection can result in underfitting or overfitting, thereby diminishing classification performance.

Most traditional parameter selection methods rely on past experience or grid search, which can lead to problems such as inappropriate parameter selection, high computational costs, and low search efficiency. As a result, these approaches do not adequately address the need for efficient parameter optimization in hyperspectral image classification [35]. To address this limitation, the Improved Octopus Optimization Algorithm (IOOA) is applied to automatically tune SVM hyperparameters, thereby enhancing classification performance. The parameter selection task is reformulated as a continuous optimization problem, enabling adaptive hyperparameter configuration through automated optimization. The effectiveness and practical applicability of the proposed algorithm are assessed by comparing its performance with other hyperparameter optimization algorithms.

### 5.2. Dataset Introduction

The effectiveness of the proposed method in hyperspectral image classification is evaluated on the Pavia University dataset [36]. This dataset was acquired by ROSIS sensors. The images have a spatial resolution of 610 × 340 pixels, contain 103 spectral bands, and feature ground truth annotations for nine land cover classes. This dataset represents a typical urban hyperspectral scene, characterized by high spatial complexity and spectral similarity among classes; as such, it is frequently used to evaluate the performance of hyperspectral image classification methods.

**Figure 7:**
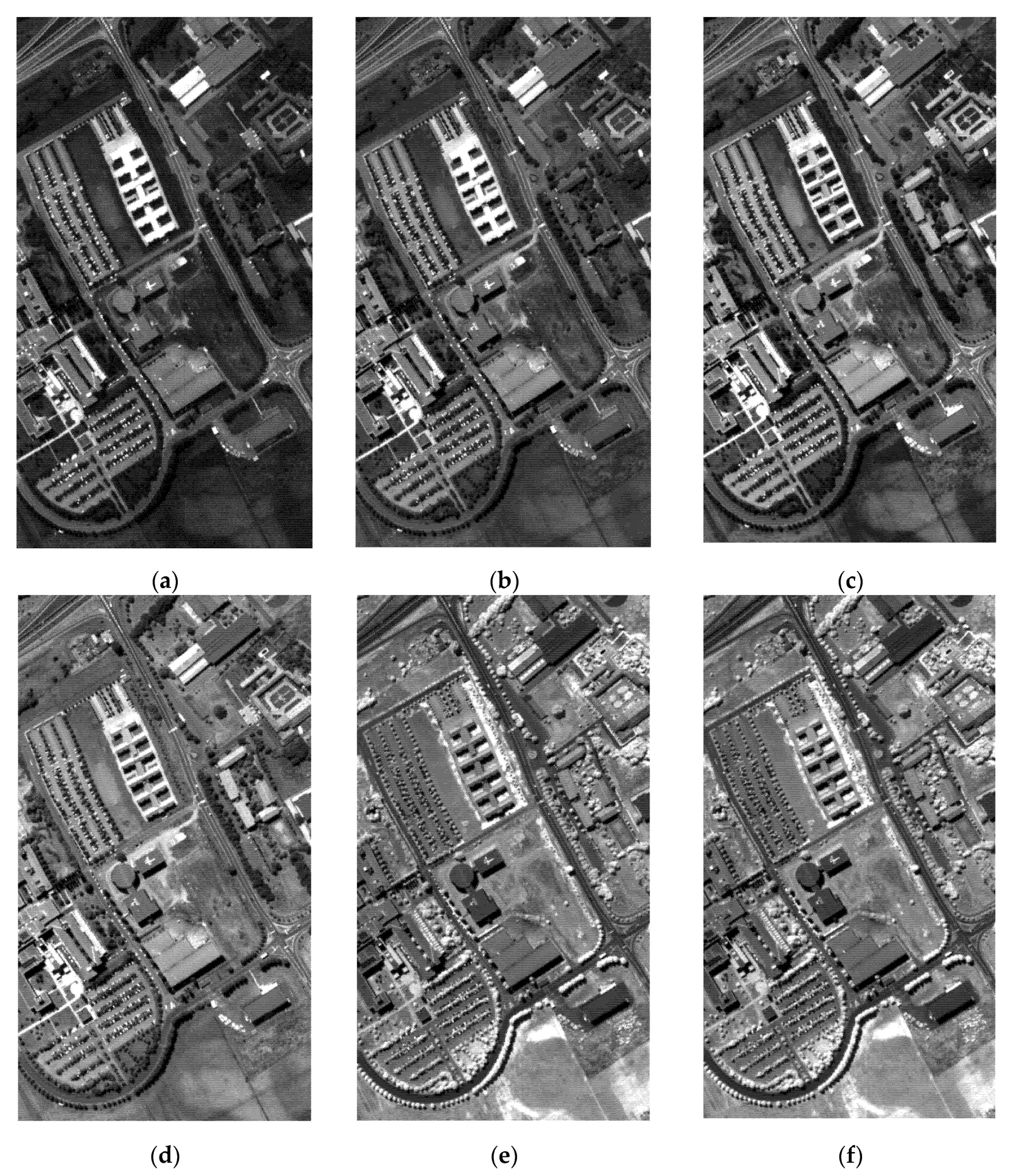
Representative spectral bands of the Pavia University dataset: (**a**) the 10th spectral band; (**b**) the 30th spectral band; (**c**) the 50th spectral band; (**d**) the 70th spectral band; (**e**) the 90th spectral band; (**f**) the 100th spectral band.

Figure 7 displays six representative spectral bands from the Pavia University dataset to provide a more intuitive understanding of the experimental data. The Pavia University dataset contains rich spectral information across multiple bands; different spectral bands can reveal the spatial structure, texture distribution, and spectral response of different objects within the same scene. Therefore, the representative bands shown help to better illustrate the spatial and spectral characteristics of the dataset, as well as the complexity of hyperspectral image classification tasks.

### 5.3. Extraction of Spatial and Spectral Features from the Pavia University Dataset

In this paper, a spatial-spectral feature extraction strategy based on principal component analysis (PCA) and extended morphological profiling (EMP) was applied to the Pavia University dataset prior to image classification. The original hyperspectral data cube was reconstructed into a two-dimensional matrix, where each row represents a pixel, and each column represents a spectral band. Next, the spectral data were normalized on a band-by-band basis, and PCA analysis was performed on the normalized data.

This paper constructs EMP features using images derived from the first three principal components to extract spatial features. The original image for each principal component was retained, and morphological opening and closing operations were performed using disk-shaped structural elements with radii of two, four, and six, respectively. Therefore, each principal component image generates seven spatial features, including one original principal component image and six morphological contour features, resulting in a total of 21 spatial features. At the same time, the first 30 principal components are retained as spectral features. Finally, the 21-dimensional EMP spatial features and the 30-dimensional PCA spectral features are combined to form a 51-dimensional spatio-spectral fusion feature vector for each pixel. Before feeding the data into the SVM classifier, the features are further standardized, and unlabeled pixels with a label of zero are removed; only labeled pixels are used for training and testing.

### 5.4. Experimental Design and Evaluation Criteria

This paper conducts experiments using the Pavia University dataset. First, this paper removed background pixels with a label value of zero, retaining only the 42,776 valid annotated samples with actual object category labels for the classification experiment. Then, all valid labeled samples were randomly divided into a training set and a test set in a 1:9 ratio; that is, 10% (approximately 4278 samples) were used for model training, and 90% (approximately 38,498 samples) were used for the final test. This approach allows us to evaluate the impact of different optimization algorithms on the ability to optimize SVM hyperparameters under conditions of limited training data, while also using a larger test dataset to assess the generalization performance of the classification model more thoroughly. In this paper, the random seed used for partitioning the external samples is set to one to ensure the reproducibility of the experiments and fairness when comparing different algorithms.

The classification model uses a support vector machine based on the radial basis function kernel, with the parameters to be optimized being the regularization parameter C and the kernel parameter γ. All algorithms were compared under a standardized experimental setup, with a population size of 30, a maximum number of iterations set to 50, and 10 independent runs. The proposed IOOA was evaluated against OOA, PSO, GWO, SSA, AOA and SCSO to verify its effectiveness.

Given that repeatedly training and validating classifiers on the full training set for each set of candidate parameters during the optimization process would entail a high computational cost, this paper employs a lightweight internal evaluation strategy during the fitness evaluation phase. Specifically, a small number of samples are randomly sampled from the training set by class to construct internal training and validation sets, and the classification error rate on the internal validation set is used as the fitness value. The internal sampling random seed is fixed at 42, and the number of samples used for internal training and validation in each class does not exceed 15. The Pavia University dataset contains nine land cover classes, so during the fitness evaluation phase, a maximum of 135 samples are used as the internal training subset and 135 samples as the internal validation subset. Both the internal training subset and the internal validation subset are drawn from the training set. After obtaining the optimal set of parameters, this paper retrains the SVM classifier using the full training set and evaluates the final classification performance on an independent test set. This configuration reduces the computational overhead of fitness calculations while effectively preserving the ability to distinguish between good and bad parameters.

Each algorithm was run independently 10 times to minimize the impact of randomness on the results. This paper uses Overall Accuracy (OA) as the primary evaluation metric, which is defined as

(34) $$OA=\frac{N_{c}}{N_{t}}\times 100\%$$

Here, $N_{c}$ represents the number of samples correctly classified in the test set, and $N_{t}$ represents the total number of test samples. At the same time, the optimal value, worst value, average value, and standard deviation of the OA for each algorithm after 10 independent runs were calculated to measure optimal performance, worst-case performance, average performance, and result stability, respectively. Additionally, the average values of the optimal parameters in logarithmic space, ${log}_{10}C$ and ${log}_{10}\gamma$, were calculated to analyze the parameter search results and stability of different algorithms.

### 5.5. Analysis of Experimental Results

As shown in Table 7, there are significant differences in the distribution of SVM parameters obtained using different optimization algorithms. The average parameters obtained by IOOA over 10 independent runs were ${log}_{10}C=2.1323$ and ${log}_{10}\gamma =1.8314$, with a corresponding average classification accuracy of 97.3731%, which was the highest among all algorithms. The average parameters for PSO and GWO are (2.3968, 1.9676) and (2.5642, 2.0462), respectively, which are quite close to those of IOOA. Their average classification accuracies are 97.3100% and 97.3271%, respectively, and their overall performance is also relatively stable.

In contrast, the parameters obtained from OOA, SSA, and SCSO searches generally tend toward the region with larger values of C and γ, with OOA’s average parameters being (3.3976, 2.4628), SSA’s average parameters being (2.9403, 2.2411), and SCSO’s average parameters being (3.0378, 2.2852); their average classification accuracies are all lower than that of IOOA. The average parameters for AOA are (1.4366, 1.7482), but the classification results show significant variation. Overall, IOOA is able to obtain reasonably optimal parameter combinations in the parameter space and strike a good balance between classification accuracy and result stability.

As shown in the results in Table 7, IOOA performs best in terms of overall performance. The best value, worst value, and average value were 97.6310%, 97.1531%, and 97.3731%, respectively. In which, both the average and the worst-case values were the highest among all algorithms, indicating that the strategy proposed in this paper not only achieves superior classification results but also exhibits good operational stability. GWO and PSO followed with average accuracies of 97.3271% and 97.3100%, respectively; OOA, SSA, and SCSO had relatively lower average accuracies.

In terms of stability, the standard deviation of IOOA is 0.001806, which is lower than that of GWO and AOA and comparable to that of PSO, SSA, and SCSO, indicating that its results are generally stable across multiple runs. Furthermore, although AOA achieved the highest single-classification accuracy of 97.7791%, its worst-case classification accuracy was only 43.6750%, and its average classification accuracy dropped to 91.7232%. This indicates that the algorithm is relatively sensitive to the initial population and the search process, resulting in insufficient stability of the results. Based on the overall best score, worst score, average score, and standard deviation, it can be concluded that IOOA demonstrates superior overall classification performance on the Pavia University dataset.

This paper uses the Wilcoxon rank-sum test to conduct pairwise comparisons of the results from 10 independent runs of IOOA and each of the comparison algorithms to test for statistical significance in the differences in classification performance among the various algorithms, with a significance level of 0.05. The results are shown in Table 8. The *p*-values obtained by comparing IOOA with OOA, SSA, and AOA were 0.001953, 0.019531, and 0.013672, respectively, all of which were less than 0.05. Furthermore, IOOA achieved a higher average classification accuracy, indicating that its performance improvement is statistically significant. The *p*-values obtained from comparisons with PSO, GWO, and SCSO were all greater than 0.05, indicating that their respective performance levels are comparable. Overall, IOOA demonstrates significant advantages over some of the comparison algorithms and remains highly competitive in the remaining comparisons.

**Figure 8:**
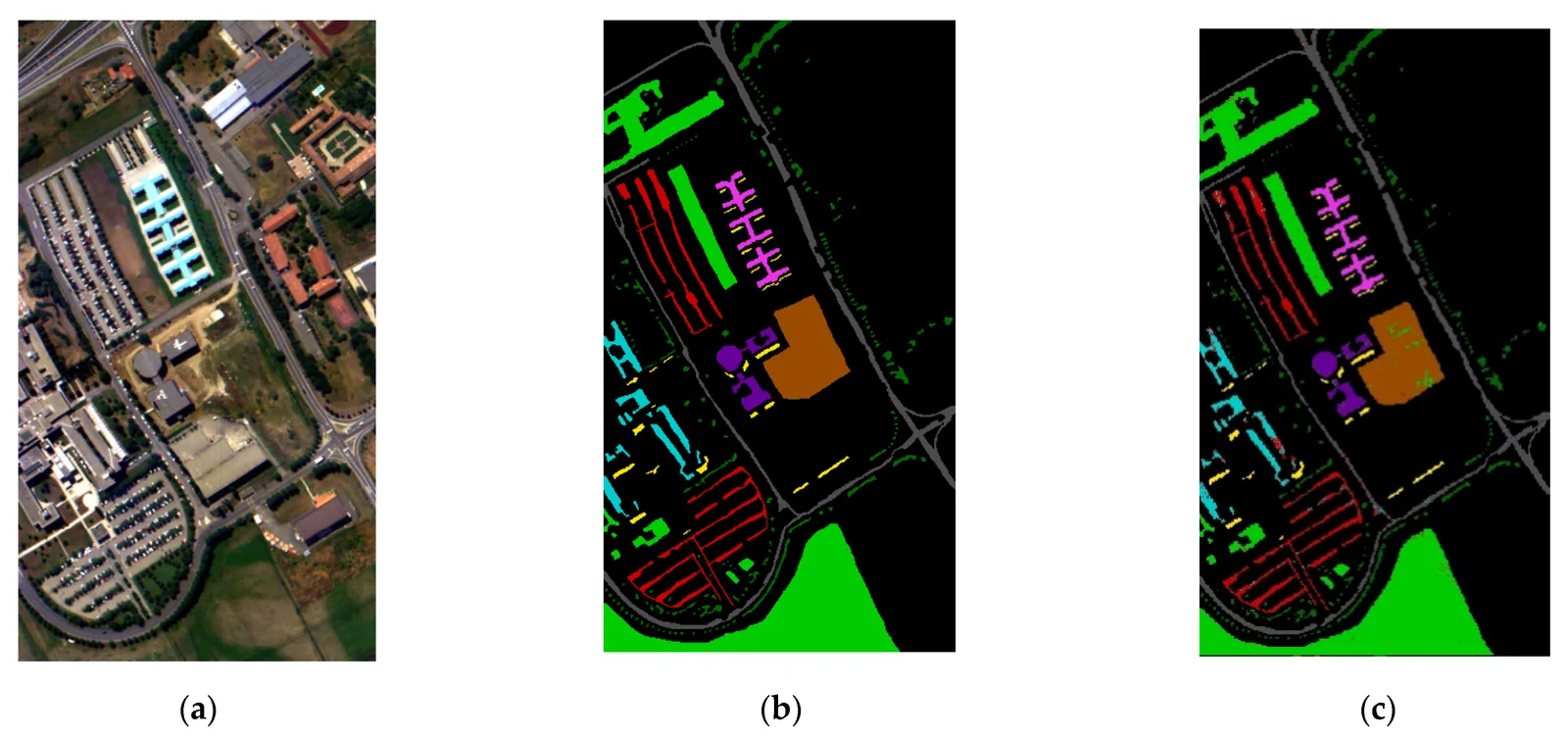

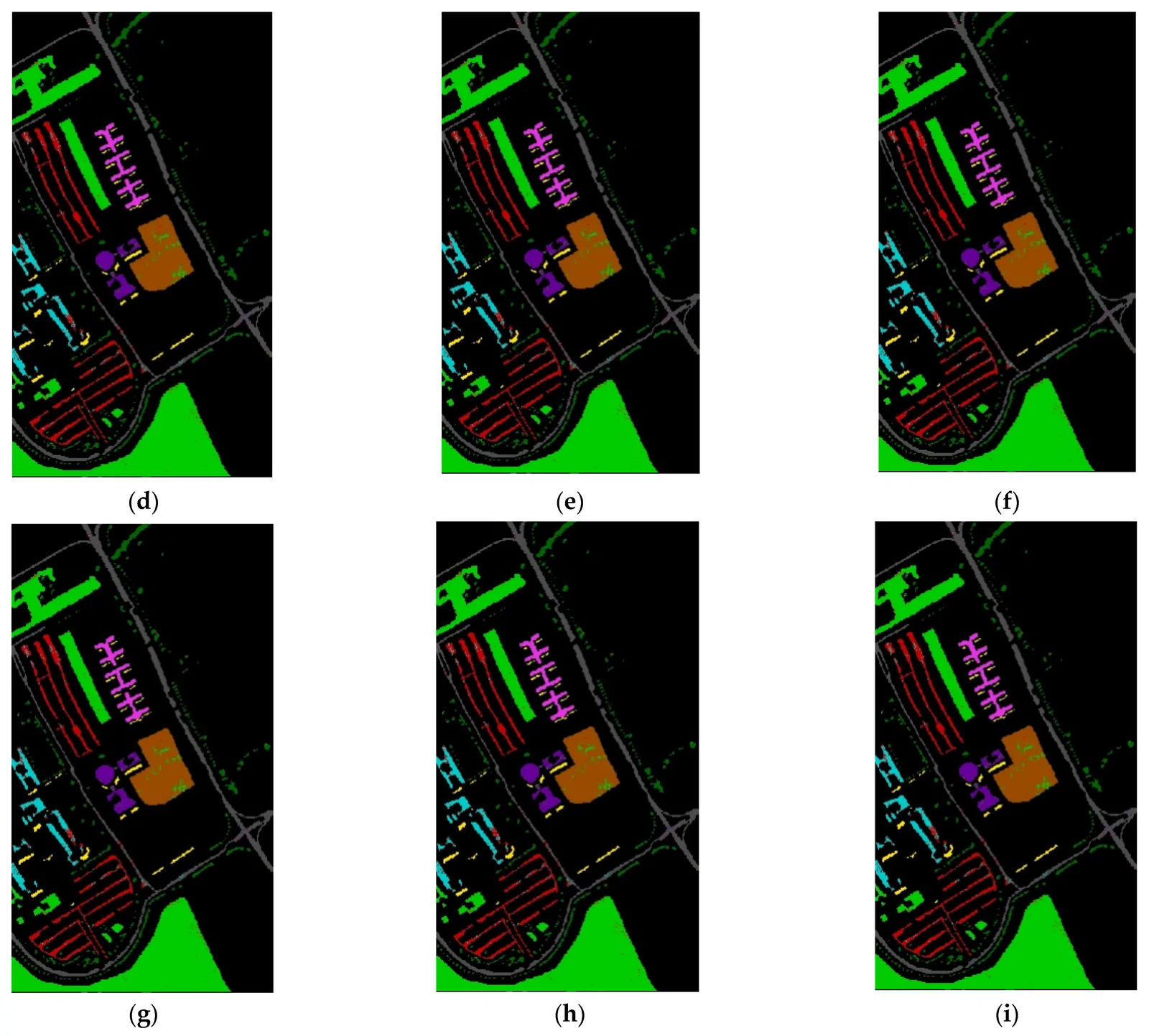
Visual comparison of the Pavia University dataset’s false-color imagery, true land-cover maps, and classification results from various algorithms: (**a**) Pavia University pseudocolor image; (**b**) Pavia University ground truth; (**c**) IOOA classification results chart, mean OA: 97.3731%; (**d**) OOA classification results chart, mean OA: 97.1453%; (**e**) PSO classification results chart, mean OA: 97.3100%; (**f**) GWO classification results chart, mean OA: 97.3271%; (**g**) SSA classification results chart, mean OA: 97.2209%; (**h**) AOA classification results chart, mean OA: 91.7232%; (**i**) SCSO classification results chart, mean OA: 97.2248%.

Figure 8 displays the pseudocolor image, the ground truth map, and the classification results for each algorithm, facilitating a visual comparison of classification performance. Different colors represent different object categories, and each color corresponds to a specific category label. Overall, all algorithms effectively recover the primary spatial distribution features of the scene, and the classification maps exhibit strong consistency with the ground truth maps across extensive regions.

The classification map produced by IOOA demonstrates superior regional integrity and boundary preservation. IOOA accurately captures the spatial distribution of strip-, block-, and linear-shaped features, with relatively few misclassified points in localized areas. These results more closely correspond to the ground truth map than those generated by other methods. In contrast, OOA, SSA, AOA and SCSO exhibit varying degrees of class overlap and local fragmentation in certain boundary regions. While the overall classification performance of PSO and GWO is comparable to that of IOOA, IOOA exceeds both in terms of local continuity and regional consistency.

Overall, IOOA demonstrates superior performance in terms of regional integrity, boundary clarity, and suppression of local misclassifications. These findings support the effectiveness of the proposed strategy for addressing the hyperparameter optimization problem in hyperspectral image classification.

### 5.6. Comparison with State-of-the-Art Methods on the Pavia University Dataset

Table 9 in this paper compares the IOOA-SVM method with several other representative hyperspectral image classification methods tested on the Pavia University dataset, in order to better evaluate the effectiveness of the proposed method. This paper selects SMLR, KSMLR, and LELM from the work of Cao et al. [37], 2D-CNN, 3D-CNN, and M3D-CNN from the work of Roy et al. [38], and SVM+PCA-20, SVM+R-PCA-30, and LightGBM+PCA-20 from the work of Ustuner [39]. These methods encompass various types of classification models, including traditional statistical classifiers, extreme learning machine-based methods, deep learning methods such as CNNs, and machine learning methods based on PCA/R-PCA-derived features. All of them have been tested on the Pavia University dataset and thus provide a useful reference for evaluating the classification performance of the IOOA-SVM method proposed in this paper.

The values in the table are based on the following: The paper by Cao et al. reports the overall accuracy (OA) for methods such as SMLR, KSMLR, and LELM using a 10% training sample setting on the Pavia University dataset; the paper by Roy et al. reports the OA for 2D-CNN and 3D-CNN, and M3D-CNN on the Pavia University dataset; Ustuner’s paper reported the OA for the 70% training sample setting on the Pavia University dataset for SVM+PCA-20, SVM+R-PCA-30, and LightGBM+PCA-20.

As shown in Table 9, the IOOA-SVM method proposed in this paper achieved the highest classification accuracy (OA = 97.37%) on the Pavia University dataset compared to other comparison algorithms. Traditional statistical learning methods (SMLR and KSMLR) are limited by their feature representation capabilities, resulting in relatively poor classification performance. LELM improves classification performance through local constraints, but its feature representation capabilities are limited, and its classification performance remains relatively low. Although deep learning-based methods (2D-CNN, 3D-CNN, and M3D-CNN) can achieve good classification results through hierarchical feature learning, their performance depends on the network architecture design and parameter configuration.

### 5.7. Scalability, Limitations, and Relevance to Digital Biomimetics

This paper is closely related to the theme of the special issue “Advances in Digital Biomimetics.” The IOOA proposed in this paper is a biomimetic optimization algorithm inspired by the behavioral mechanisms of octopuses. Its design is based on behaviors such as the octopus’s search for potential prey, evasion of predators, hunting attacks, and mating. This paper digitally models these biological behaviors and further optimizes them to construct a biomimetic optimization algorithm for solving complex parameter optimization problems. This paper applies IOOA to hyperspectral image classification tasks, demonstrating the integration of biomimetic optimization methods with machine learning and intelligent remote sensing image analysis. Therefore, this paper is closely related to the theme of the Special Issue “Advances in Digital Biomimetics.”

This paper discusses the scalability of the proposed method from the perspectives of optimization algorithms and hyperspectral classification applications. At the algorithm optimization level, IOOA was validated on the CEC2017 test function set, which includes unimodal functions, simple multimodal functions, hybrid functions, and composition functions, and can, to some extent, reflect the algorithm’s optimization capabilities across search landscapes of varying complexity. At the application level, the hyperparameters optimized in this paper are those of the RBF-SVM; they do not increase with the number of bands or pixels in the hyperspectral imagery. Therefore, the IOOA-SVM framework can be used to train SVMs for other hyperspectral datasets by substituting the dataset while following the same optimization process.

The hyperspectral classification experiments in this paper are primarily based on the Pavia University dataset, comprising extensive pixel samples and a variety of typical urban land cover classes. Given the dataset’s highly heterogeneous spatial distribution and the spectral similarities and boundary blurring between different classes, it effectively reflects the characteristics of complex urban hyperspectral classification tasks to a certain extent. The experimental results in this paper demonstrate that the proposed IOOA-SVM framework has a certain degree of applicability and application potential in this typical hyperspectral scenario. However, a single dataset is still insufficient to fully demonstrate the method’s generalizability across different sensors, different land cover classes, and larger-scale remote sensing scenarios. Future research will continue to validate the scalability, robustness, and computational efficiency of the proposed method using additional hyperspectral datasets, such as those from Indian Pines and Salinas.

## 6. Conclusions

An Improved Octopus Optimization Algorithm (IOOA) based on multi-strategy coordination is introduced. The algorithm employs an elite backpropagation-based initialization strategy to increase population diversity and improve coverage of the search space, thereby increasing the probability of generating high-quality initial solutions. A multi-stage, nonlinear, adaptive parameter control mechanism enables dynamic adjustment throughout different search phases, effectively balancing global exploration and local exploitation. The integration of an elite-guided differential variation strategy further improves performance during late-stage development and fine-tuning. Additionally, the combination of a Lévy flight restart mechanism based on stagnation monitoring with a stable boundary-handling strategy strengthens the algorithm’s capacity to escape local optima and increases overall search robustness.

Experimental results on the CEC2017 test function set demonstrate that IOOA outperforms OOA and other comparison algorithms in convergence accuracy, convergence speed, and stability. These findings confirm that the proposed enhancements improve overall optimization performance. Furthermore, IOOA is applied to the hyperparameter optimization problem for hyperspectral image classification on the Pavia University dataset using the IOOA-SVM parameter optimization framework. This approach identifies optimal parameter combinations, resulting in higher classification accuracy and superior optimization outcomes.

In summary, IOOA addresses the performance limitations of the original OOA in complex optimization scenarios and demonstrates both feasibility and effectiveness in engineering applications, particularly in hyperparameter optimization for hyperspectral image classification. Future research may extend this algorithm to high-dimensional, dynamic, and multi-objective optimization problems, as well as apply it to a broader range of hyperspectral datasets, classification models, and practical remote sensing tasks, thereby increasing its versatility and engineering value.

## Figures and Tables

**Table 1:** Main improvements of IOOA over the original OOA.

Improvement Strategies	Issues Addressed	Main Functions
Elite-based Opposition-based Learning Population Initialization Strategy	Insufficient random initialization and limited population diversity	Improving the quality of initial solutions and search coverage
Multi-stage Nonlinear Adaptive Parameter Control Mechanism	Insufficient capability for fine-grained local search	Enhance global exploration in the early stages, and enhance local exploitation in the later stages
	PSO	$\omega_{max}=0.9,\omega_{min}=0.2,c_{1}=c_{2}=2$
Elite-Guided Differential Mutation Local Search Strategy	Insufficient capability for fine-grained local search	Improve development capability and convergence accuracy in the vicinity of high-quality regions
	SSA	$C_{1}=2exp\left(-{\left(\frac{4t}{T}\right)}^{2}\right)$
	SCSO	$S=2,rg=2-\frac{2t}{T}$
A Lévy Flight Restart Mechanism Based on Stagnation Monitoring and a Stable Boundary Handling Strategy	It is prone to falling into local optima, and out-of-bound solutions affect search stability	Enhance global exploration in the early stages, and enhance local exploitation in the later stages

**Table 2:** Algorithm parameter settings.

	Algorithm	Core Parameter Settings
Propose Algorithm	IOOA	pBestRate=0.2,elite_keep_rate=0.15, $\;TF_{DE}^{0}=0.7,{CR}_{0}=0.9$
Benchmark Algorithm	OOA	$pl=12,wdamp=1.5,\beta_{1,2,3}=\left[0.5,0.25,0.75\right],$ φ=0.75,u=0.5,μ=1.0
	PSO	$\omega_{max}=0.9,\omega_{min}=0.2,c_{1}=c_{2}=2$
	GWO	$a=2-\frac{2t}{T}$
	SSA	$C_{1}=2exp\left(-{\left(\frac{4t}{T}\right)}^{2}\right)$
	AOA	${MOP}_{max}=1,{MOP}_{min}=0.2,\alpha =5,\mu =0.499$
	SCSO	$S=2,rg=2-\frac{2t}{T}$
	DE	$F_{min}=0.2,F_{max}=0.8,CR=0.2$
	L-SHADE	$H_{m}=5,M_{F}^{0}=M_{CR}^{0}=0.5,\sigma =0.1,H_{r}=6$
Common Parameters	ALL	$N=30,T_{max}=500,Dim=30,Runs=30$

**Table 3:** CEC2017 test function set.

	NO.	Functions	$F_{i}^{*}\;=\;F_{i}\left(x^{*}\right)$
Unimodal Functions	1	Shifted and Rotated Bent Cigar Function	100
	3	Shifted and Rotated Zakharov Function	300
Simple Multimodal Functions	4	Shifted and Rotated Rosenbrock’s Function	400
	5	Shifted and Rotated Rastrigin’s Function	500
	6	Shifted and Rotated Expanded Scaffer’s F6 Function	600
	7	Shifted and Rotated Lunacek Bi-Rastrigin Function	700
	8	Shifted and Rotated Non-Continuous Rastrigin’s Function	800
	9	Shifted and Rotated Lévy Function	900
	10	Shifted and Rotated Schwefel’s Function	1000
Hybrid Functions	11	Hybrid Function 1	1100
	12	Hybrid Function 2	1200
	13	Hybrid Function 3	1300
	14	Hybrid Function 4	1400
	15	Hybrid Function 5	1500
	16	Hybrid Function 6	1600
	17	Hybrid Function 7	1700
	18	Hybrid Function 8	1800
	19	Hybrid Function 9	1900
	20	Hybrid Function 10	2000
Composition Functions	21	Composition Function 1	2100
	22	Composition Function 2	2200
	23	Composition Function 3	2300
	24	Composition Function 4	2400
	25	Composition Function 5	2500
	26	Composition Function 6	2600
	27	Composition Function 7	2700
	28	Composition Function 8	2800
	29	Composition Function 9	2900
	30	Composition Function 10	3000
Search Range: ${\left[-100,\;100\right]}^{D}$			

**Table 4:** Comparison of optimization results for various algorithms on the CEC2017 function.

Func		IOOA	OOA [17]	PSO [10]	GWO [11]	SSA [12]	AOA [13]	SCSO [14]	DE [15]	L-SHADE [16]
F1	Mean	**1183.73604**	2833.591895	2,107,418,877	2,837,814,424	6293.896941	5,284,704,3629	7,457,924,990	1,180,202.852	134,474,741.5
	Std	**1604.59006**	3022.37047	2,179,630,115	1,693,274,132	5975.8138	9,161,188,017	3,517,180,199	757,702.599	545,641,204
	Best	**100.3675625**	111.7086127	2210.38967	125,760,579	222.8360493	33,495,046,884	1,085,711,544	167,714.9914	4976.862255
	Worst	**6496.319252**	12,517.13322	9,292,975,868	7,507,540,227	19,717.79847	69,387,590,674	19,311,377,619	2,873,840.495	2,975,688,162
F3	Mean	**327.802181**	457.132385	70,105.7966	65,343.8065	68,340.4504	83,729.7388	56,136.773	165,064.815	240,104.432
	Std	**37.5665662**	101.503146	23,005.9671	11,036.3054	25,853.824	6925.94263	11,471.8117	25,484.705	85,350.3897
	Best	**301.565162**	328.823174	37,273.086	48,312.2979	28,052.9913	69,633.5014	35,513.2484	112,729.678	46,301.7278
	Worst	**506.91005**	740.111652	112,743.807	101,696.946	162,735.067	95,374.7981	75,317.7769	210,487.296	403,929.59
F4	Mean	**4** **67.765446**	491.143457	631.418061	645.609146	514.940812	14,666.1301	1133.48699	521.075253	470.777509
	Std	25.0919121	26.6594055	187.857434	108.494666	25.2441144	5515.10407	597.276905	**18.1571502**	62.8857452
	Best	**400.0** **39215**	400.132469	476.230212	539.502945	464.956819	2931.23708	538.563916	494.807841	426.557871
	Worst	**508.721404**	538.124725	1144.67865	1020.74898	593.811452	30,688.3578	3535.9686	557.949152	670.310734
F5	Mean	**528.488973**	535.0586	590.117119	625.398953	683.054761	892.15041	762.723672	688.50348	680.05753
	Std	**5.94960039**	9.62172944	27.1287061	38.3598298	56.8346533	32.0207221	41.3284961	10.4584145	43.0423541
	Best	516.914294	**515.919335**	548.136671	558.256843	579.59653	817.058353	682.468188	671.138045	591.87726
	Worst	**545.768056**	555.717618	669.630389	735.36557	808.434034	963.42222	866.132398	714.491414	756.182142
F6	Mean	601.951274	606.203946	606.45196	613.723784	653.617625	680.338097	661.753842	**600.165675**	604.590739
	Std	0.904295817	1.972999345	3.275404313	4.416045317	10.31397956	7.426552423	9.082882686	**0.044903136**	10.05630862
	Best	600.775442	602.206561	601.641791	607.112846	634.659924	664.658804	641.890442	600.09869	**600.001328**
	Worst	604.401254	610.63054	614.854061	625.772982	678.832689	695.738081	675.863206	**600.292029**	647.917979
F7	Mean	**786.107286**	827.344667	857.130256	927.39635	950.716401	1400.89883	1135.09753	933.555339	934.416546
	Std	16.5371593	29.9968862	54.8085956	51.7979369	68.5934251	57.0176945	82.9728205	**11.7001838**	55.78933
	Best	**762.527769**	784.888515	772.600193	845.618644	859.520656	1271.30723	1013.41308	903.117113	826.09515
	Worst	**840.177598**	903.554277	1039.61964	1053.67375	1190.15572	1484.51091	1349.64727	951.840318	1089.44425
F8	Mean	**834.72404**	849.94998	885.753832	906.422125	944.760444	1116.92071	996.800086	995.862942	989.306165
	Std	**7.89225455**	15.8189777	25.5420722	34.4696353	36.2382426	26.0942468	31.2864565	13.0167585	35.8470159
	Best	**820.894135**	826.863887	846.793663	861.11753	870.641961	1075.21125	929.703612	970.882731	931.759855
	Worst	**848.752948**	880.591508	944.402627	1043.48282	1007.94443	1174.4903	1056.01507	1018.11906	1068.44747
F9	Mean	**9** **59.628118**	1101.1608	2081.77756	3073.0592	5632.06813	7902.78855	6770.1109	2125.81393	2346.16738
	Std	**25.3031**	99.3320139	2058.19847	1820.15292	1420.25668	1440.71851	1408.05868	536.214976	2379.2931
	Best	916.240444	953.156956	923.409309	1385.60955	3450.83692	5672.26225	4001.0478	1421.54285	**900.** **195193**
	Worst	**1003.25186**	1485.47873	10,655.4672	9134.00639	8952.02075	11,424.3302	10,655.4672	3872.57991	12,536.9676
F10	Mean	**3968.61985**	4517.20403	4669.13924	5141.35016	5201.82967	7848.12486	6081.10984	7724.13099	8503.80347
	Std	588.673077	532.04454	879.154338	1493.61905	631.563127	487.859499	636.372334	**258.624065**	895.797318
	Best	**2697.62722**	3402.95233	2882.60112	3752.8362	4001.67232	6893.74677	4715.7532	7037.53051	5138.43571
	Worst	**5218.9283**	5552.88942	6662.07076	9475.16547	6332.48508	8991.21938	7099.38444	8206.97949	9629.24062
F11	Mean	**1163.57765**	1195.57995	1299.31097	2437.07973	1452.25141	10,036.7481	2788.12513	1975.07458	1620.34508
	Std	**29.7218481**	35.6915466	65.7738096	1115.77489	115.728666	2785.16448	1403.90245	608.55323	754.88887
	Best	**1113.94074**	1141.92519	1187.15334	1344.52682	1250.68408	5400.09817	1288.81338	1420.35112	1160.69207
	Worst	**1222.98191**	1282.86736	1480.11696	4742.02931	1846.88189	16,459.6469	6215.18273	4240.03865	4427.88664
F12	Mean	**37,993.3822**	208,929.0524	41,425,762.7	98,715,203.94	35,174,990.24	14,307,473,875	383,742,429.8	34,810,714.17	66,463,933.01
	Std	**31,833.6914**	217,566.415	72,720,797.5	107,556,956	29,050,987.6	4,137,616,598	493,937,178	16,583,646.2	74,173,075
	Best	**6136.42816**	15,481.3776	289,455.347	9,332,100.09	2,717,265.14	4,866,095,536	25,130,408.3	12,937,734.1	312,250.198
	Worst	**147,212.2792**	1,013,602.596	220,473,801.8	454,495,508.8	119,978,043.2	22,093,953,779	2,036,205,856	78,700,158.54	251,987,464.2
F13	Mean	**9896.43169**	18,590.62128	3,471,120.582	15,680,767.8	127,294.1542	12,872,090,588	87,214,395.39	4,554,952.825	5,967,459.033
	Std	**8070.36609**	10,491.6411	13,021,726.3	33,605,334.2	97,058.439	5,457,744,514	169,275,039	3,754,934.71	12,538,926.1
	Best	**1353.90266**	3626.07629	3947.076	82,298.6703	12,433.1043	3,978,627,058	32,313.5554	621,169.084	40,893.8376
	Worst	**32,012.45046**	38,588.05378	71,682,894.49	139,854,338.2	407,803.1398	25,645,742,045	712,170,951.4	17,778,260.53	64,084,770.93
F14	Mean	**1706.12149**	1791.10161	182,688.686	644,648.532	167,372.691	5,546,426.94	614,305.594	414,600.902	113,432.955
	Std	381.766974	**318.275819**	411,150.086	553,448.924	251,940.638	9,574,823.98	798,228.486	223,497.231	227,090.878
	Best	**1464.23915**	1494.65645	7182.50713	4230.9804	5382.28166	115,077.252	9169.27649	100,630.144	1605.6162
	Worst	3238.92914	**2862.26723**	1,672,791.59	1,879,105.67	1,249,206.05	50,373,655	3,563,386.78	1,186,235.18	1,172,580.03
F15	Mean	**2134.08789**	2582.57161	24,775.5431	5,063,342.46	68,024.9884	454,103,888	4,929,962.01	750,316.176	789,068.529
	Std	**905.611727**	993.237734	29,076.3365	18,081,361	37,417.6651	655,157,645	14,285,016.8	767,082.679	1,322,211.19
	Best	1529.93443	**1524.48166**	2425.00569	34,080.9001	9411.33469	17,934.4534	21,646.3628	63,358.4657	3655.83122
	Worst	**4873.59217**	5240.22405	102,373.575	80,352,966.1	165,694.068	2,712,247,231	70,432,433.2	3,923,022.78	4,701,028.2
F16	Mean	**2017.2477**	2218.56519	2625.1914	2645.58516	3035.46589	5994.38486	3232.31762	3002.09095	3442.41089
	Std	241.335618	251.465098	289.050806	256.935354	373.887158	1951.97282	480.954928	**145.817635**	333.641249
	Best	**1605.80541**	1737.3505	2041.5324	2135.91419	2118.26607	3775.58608	2162.16669	2745.19097	2566.02277
	Worst	**2613.32659**	2673.91564	3182.86905	3446.68513	3743.64945	11,596.2958	4503.00942	3300.12015	3982.98825
F17	Mean	**1786.73503**	1882.73592	2146.37001	2119.17217	2384.34353	5252.20708	2423.57233	2193.5077	2450.36172
	Std	89.6253616	137.755491	207.125773	205.796641	306.657625	2371.45194	236.280016	**88.0853557**	254.852401
	Best	**1707.45101**	1735.83711	1818.93917	1872.12345	1844.75951	2791.58281	2007.15552	2015.85791	1903.96181
	Worst	**2081.02191**	2253.11128	2520.88089	2762.20532	3226.18403	13,486.7868	2823.49678	2316.67695	3076.92193
F18	Mean	**18,298.4661**	31,292.6595	921,615.586	2,358,039.32	1,973,182.72	42,585,619.9	3,023,931.19	3,318,886.47	5,656,236.35
	Std	9459.50155	**9105.12947**	929,936.622	2,526,244.11	2,374,513.59	29,512,627.8	3,300,916.74	1,815,362.63	5,795,241.56
	Best	**5297.32227**	11,818.5787	103,295.655	63,123.4818	135,334.794	2,200,018.48	47,908.8178	1,106,189.55	118,310.002
	Worst	**44,272.8892**	59,782.24	3,550,973.26	9,454,008.99	10,678,519.8	133,049,769	11,203,439.7	9,384,648.93	23,046,037.8
F19	Mean	**4529.80092**	5862.8789	35,363.8268	2,498,179.44	5,708,997.54	531,360,335	4,071,311.76	671,923.213	338,197.143
	Std	**2971.3859**	2984.25777	58,062.0546	5,510,423.71	3,939,766.59	401,022,106	9,388,254.85	745,182.158	930,683.159
	Best	**1909.80817**	2271.44354	2119.79841	20,128.6941	66,201.6569	3,103,281.07	37,503.5711	45,287.5798	2046.89278
	Worst	**12,665.8706**	16,823.3198	177,950.588	26,654,377.5	15,757,195.3	1,336,558,384	48,687,162.3	333,2013.51	4,894,420
F20	Mean	**2183.40643**	2330.01604	2361.21402	2469.08102	2636.27409	2857.90436	2754.17191	2512.79847	2680.0709
	Std	**85.8401373**	174.067149	173.976797	152.425732	226.117291	176.733713	221.304594	109.241617	233.196066
	Best	**2037.64242**	2155.57659	2055.18047	2253.06451	2208.16375	2557.12129	2245.0461	2328.83967	2083.00726
	Worst	**2370.03792**	2944.53038	2820.06374	2843.25891	3088.88094	3245.95426	3094.08802	2781.01533	3239.40386
F21	Mean	**232** **7.90553**	2335.82407	2414.27672	2403.94145	2453.47777	2689.4527	2529.46703	2497.93094	2494.91898
	Std	**8.05453908**	9.55153277	35.7241643	34.5320279	42.2835181	51.0108022	42.2256904	12.6003296	49.2912746
	Best	**2312.44412**	2321.43254	2347.7175	2366.27381	2380.38496	2562.24105	2459.93696	2473.93336	2390.70618
	Worst	**2342.85777**	2356.47736	2513.16277	2533.71128	2548.26845	2773.74556	2619.17834	2520.79665	2579.77556
F22	Mean	**2300.88394**	2301.07132	4808.71772	5397.45753	4808.31338	8808.97456	4667.37581	5739.29189	9375.91021
	Std	**1.54838717**	1.58940379	1744.28639	1889.9579	2406.14805	865.027874	1437.48415	1890.21773	1563.10473
	Best	**2300**	2300.0001	2300.59393	2443.32809	2300.01307	6807.65127	2889.41533	3311.21471	2572.85302
	Worst	2304.90266	**2304.50578**	7580.25963	7826.23685	7653.91164	10,151.6711	7708.1659	9485.09623	10,931.1829
F23	Mean	**2681.20713**	2689.473	2920.28843	2789.70006	2796.4574	3544.06731	2934.70446	2839.40303	2855.90264
	Std	**11.0873425**	13.9653277	91.3714819	50.4506796	55.6562656	165.483553	58.3433092	11.2967058	58.6120585
	Best	**2661.34758**	2663.87805	2768.60539	2699.09333	2706.22101	3225.53143	2810.86637	2819.66152	2688.90391
	Worst	**2710.32954**	2718.14156	3165.98676	2906.79356	2971.70038	3822.38887	3046.8625	2858.94455	2933.78741
F24	Mean	**28** **49.94941**	2860.50864	3111.36365	2962.80372	2941.49951	3991.81011	3109.43919	3039.15705	3054.09045
	Std	**12.739388**	13.629415	113.636995	63.6004953	36.4567515	262.173064	69.1416901	13.2982566	62.2073485
	Best	**28** **30.67317**	2832.589	2946.26047	2876.55075	2885.27688	3533.80745	2985.66747	3007.43167	2915.1325
	Worst	**28** **81.46136**	2888.95811	3319.86932	3093.46688	3086.66087	4363.48093	3236.11038	3068.85021	3220.47891
F25	Mean	**2895.70841**	2908.20297	2918.84317	3012.5066	2954.84078	5946.56304	3235.94822	2898.82448	2912.59766
	Std	17.2463814	21.1117109	35.4258171	44.6301243	42.4028776	1018.5579	194.09611	**7.56715288**	45.5354179
	Best	2883.47906	2887.15892	2888.58111	2927.60752	2893.66605	4298.78786	3012.40471	2889.53144	**2881.19426**
	Worst	2940.87415	2945.98438	3045.42658	3136.34124	3053.70185	7687.05597	3784.15544	**2917.94487**	3100.47961
F26	Mean	**3862.64627**	3906.84973	4914.27661	4903.10166	5074.86247	10,772.4998	7022.79488	5541.86485	5396.99811
	Std	303.886452	627.847336	621.428273	340.610875	1044.16665	1154.79813	1074.76431	**146.860536**	520.725543
	Best	2900	**2800.00154**	3686.44419	4374.26087	2800.00899	8463.93255	4394.59512	5236.08003	3860.44458
	Worst	**4372.13586**	5212.02991	6281.40689	5573.73494	6540.95976	13,126.0124	9511.08751	5779.50011	6273.52374
F27	Mean	3222.31209	3239.25168	3292.13693	3275.13173	3282.13919	4727.49119	3418.2741	3229.99702	**3200.00701**
	Std	11.8879279	14.1909525	55.5245253	40.4702415	43.9624466	375.817495	94.5785809	4.7593803	**0.00012151**
	Best	3204.08763	3220.10856	3227.1056	3228.32911	3223.67109	4116.17268	3283.84279	3221.53236	**3200.00672**
	Worst	3255.36253	3272.27045	3424.10193	3417.91211	3392.7282	5716.41433	3633.70728	3240.97969	**3200.00721**
F28	Mean	**3170.78482**	3210.93279	3466.36063	3532.73981	3309.79941	7013.71017	3777.4942	3299.71269	3300.00133
	Std	49.4773716	12.1203571	267.911797	151.615487	42.0402395	922.337533	362.565766	27.0127315	**0.03149793**
	Best	**3100.00145**	3194.80686	3230.12706	3315.62849	3252.3989	5674.75672	3263.03116	3275.34726	3299.83456
	Worst	**3240.186**	3252.969	4404.84455	4010.08339	3431.32135	9246.73224	5174.49099	3409.63088	3300.00727
F29	Mean	**3470.39362**	3564.63501	3765.44326	3987.04762	4325.27985	7112.0637	4612.94066	4214.4198	4290.66395
	Std	**108.419424**	165.932002	177.846805	249.180927	276.243096	1194.71285	372.084737	149.246828	453.426723
	Best	3311.52976	3356.32138	3444.29958	3526.81754	3791.90144	4896.98897	3873.36622	3932.09948	**3157.91467**
	Worst	**3740.81855**	3955.89431	4142.18949	4687.94935	4958.0721	9775.68742	5289.07131	4446.47471	5105.36679
F30	Mean	**6899.52664**	7831.62262	625,376.311	12,378,673	12,526,953.6	2,465,036,359	18,800,164.2	506,416.825	162,854.284
	Std	**1169.78279**	1278.01894	1,494,026.67	9133,772.91	7,699,981.09	1,260,307,904	17,003,348.1	429,457.282	333,465.429
	Best	5513.3228	5805.16067	9126.81383	1,513,033.62	1,191,398.85	285,640,236	479,035.108	94,953.7165	**4086.82225**
	Worst	**9309.68536**	11,313.7142	7,109,519.56	36,194,567	31,310,429.3	4,917,973,313	62,232,940.8	2,112,017.62	1,549,209.75

The bold black data points indicate the best results within each set of experiments.

**Table 5:** Wilcoxon rank-sum test *p*-value (IOOA vs. other algorithms).

Function	OOA [17]	PSO [10]	GWO [11]	SSA [12]	AOA [13]	SCSO [14]	DE [15]	L-SHADE [16]
F1	0.001766564	6.06576 × 10^−11^	3.01986 × 10^−11^	3.32415 × 10^−6^	3.01986 × 10^−11^	3.01986 × 10^−11^	3.01986 × 10^−11^	3.33839 × 10^−11^
F3	1.41098 × 10^−9^	3.01986 × 10^−11^	3.01986 × 10^−11^	3.01986 × 10^−11^	3.01986 × 10^−11^	3.01986 × 10^−11^	3.01986 × 10^−11^	3.01986 × 10^−11^
F4	0.000158461	6.72195 × 10^−10^	3.01986 × 10^−11^	2.0338 × 10^−9^	3.01986 × 10^−11^	3.01986 × 10^−11^	8.9934 × 10^−11^	0.387099778
F5	0.004856016	3.01986 × 10^−11^	3.01986 × 10^−11^	3.01986 × 10^−11^	3.01986 × 10^−11^	3.01986 × 10^−11^	3.01986 × 10^−11^	3.01986 × 10^−11^
F6	1.20567 × 10^−10^	3.82489 × 10^−9^	3.01986 × 10^−11^	3.01986 × 10^−11^	3.01986 × 10^−11^	3.01986 × 10^−11^	3.01986 × 10^−11^	0.016954881
F7	3.64589 × 10^−8^	1.85673 × 10^−9^	3.01986 × 10^−11^	3.01986 × 10^−11^	3.01986 × 10^−11^	3.01986 × 10^−11^	3.01986 × 10^−11^	3.33839 × 10^−11^
F8	4.08395 × 10^−5^	4.07716 × 10^−11^	3.01986 × 10^−11^	3.01986 × 10^−11^	3.01986 × 10^−11^	3.01986 × 10^−11^	3.01986 × 10^−11^	3.01986 × 10^−11^
F9	6.72195 × 10^−10^	2.37682 × 10^−7^	3.01986 × 10^−11^	3.01986 × 10^−11^	3.01986 × 10^−11^	3.01986 × 10^−11^	3.01986 × 10^−11^	0.022360148
F10	0.000691252	0.001114256	1.99628 × 10^−5^	1.3111 × 10^−8^	3.01986 × 10^−11^	8.99341 × 10^−11^	3.01986 × 10^−11^	3.68973 × 10^−11^
F11	0.000811998	2.1544 × 10^−10^	3.01986 × 10^−11^	3.01986 × 10^−11^	3.01986 × 10^−11^	3.01986 × 10^−11^	3.01986 × 10^−11^	1.85673 × 10^−9^
F12	1.25408 × 10^−7^	3.01986 × 10^−11^	3.01986 × 10^−11^	3.01986 × 10^−11^	3.01986 × 10^−11^	3.01986 × 10^−11^	3.01986 × 10^−11^	3.01986 × 10^−11^
F13	0.000620265	9.83289 × 10^−8^	3.01986 × 10^−11^	1.95678 × 10^−10^	3.01986 × 10^−11^	3.01986 × 10^−11^	3.01986 × 10^−11^	3.01986 × 10^−11^
F14	0.015014133	3.01986 × 10^−11^	3.01986 × 10^−11^	3.01986 × 10^−11^	3.01986 × 10^−11^	3.01986 × 10^−11^	3.01986 × 10^−11^	8.10136 × 10^−10^
F15	0.022360148	3.15889 × 10^−10^	3.01986 × 10^−11^	3.01986 × 10^−11^	3.01986 × 10^−11^	3.01986 × 10^−11^	3.01986 × 10^−11^	5.49405 × 10^−11^
F16	0.003033948	3.82489 × 10^−9^	7.38029 × 10^−10^	1.09367 × 10^−10^	3.01986 × 10^−11^	4.97517 × 10^−11^	3.01986 × 10^−11^	3.33839 × 10^−11^
F17	0.000300589	1.17374 × 10^−9^	6.72195 × 10^−10^	1.09367 × 10^−10^	3.01986 × 10^−11^	3.68973 × 10^−11^	4.97517 × 10^−11^	4.50432 × 10^−11^
F18	2.67842 × 10^−6^	3.01986 × 10^−11^	3.01986 × 10^−11^	3.01986 × 10^−11^	3.01986 × 10^−11^	3.01986 × 10^−11^	3.01986 × 10^−11^	3.01986 × 10^−11^
F19	0.011227764	0.003848068	3.01986 × 10^−11^	3.01986 × 10^−11^	3.01986 × 10^−11^	3.01986 × 10^−11^	3.01986 × 10^−11^	2.57212 × 10^−7^
F20	1.86085 × 10^−6^	2.13273 × 10^−5^	1.41098 × 10^−9^	3.15889 × 10^−10^	3.01986 × 10^−11^	1.61323 × 10^−10^	4.50432 × 10^−11^	5.07231 × 10^−10^
F21	0.001679756	3.01986 × 10^−11^	3.01986 × 10^−11^	3.01986 × 10^−11^	3.01986 × 10^−11^	3.01986 × 10^−11^	3.01986 × 10^−11^	3.01986 × 10^−11^
F22	0.000200581	6.69552 × 10^−11^	3.01986 × 10^−11^	2.57212 × 10^−7^	3.01986 × 10^−11^	3.01986 × 10^−11^	3.01986 × 10^−11^	3.01986 × 10^−11^
F23	0.008314609	3.01986 × 10^−11^	3.68973 × 10^−11^	3.68973 × 10^−11^	3.01986 × 10^−11^	3.01986 × 10^−11^	3.01986 × 10^−11^	4.97517 × 10^−11^
F24	0.001952677	3.01986 × 10^−11^	3.01986 × 10^−11^	3.01986 × 10^−11^	3.01986 × 10^−11^	3.01986 × 10^−11^	3.01986 × 10^−11^	3.01986 × 10^−11^
F25	0.001766564	1.09069 × 10^−5^	4.50432 × 10^−11^	4.1825 × 10^−9^	3.01986 × 10^−11^	3.01986 × 10^−11^	0.000238848	0.022360148
F26	0.008684371	5.53286 × 10^−8^	3.01986 × 10^−11^	5.46203 × 10^−6^	3.01986 × 10^−11^	3.01986 × 10^−11^	3.01986 × 10^−11^	1.77691 × 10^−10^
F27	3.83494 × 10^−6^	8.10136 × 10^−10^	7.38029 × 10^−10^	9.7555 × 10^−10^	3.01986 × 10^−11^	3.01986 × 10^−11^	6.76501 × 10^−5^	3.01986 × 10^−11^
F28	0.000811998	3.33839 × 10^−11^	3.01986 × 10^−11^	3.01986 × 10^−11^	3.01986 × 10^−11^	3.01986 × 10^−11^	3.01986 × 10^−11^	3.01986 × 10^−11^
F29	0.01563812	8.48477 × 10^−9^	3.15889 × 10^−10^	3.01986 × 10^−11^	3.01986 × 10^−11^	3.01986 × 10^−11^	3.01986 × 10^−11^	1.41098 × 10^−9^
F30	0.000952074	4.50432 × 10^−11^	3.01986 × 10^−11^	3.01986 × 10^−11^	3.01986 × 10^−11^	3.01986 × 10^−11^	3.01986 × 10^−11^	7.59915 × 10^−7^
+/NaN/−	29/0/0	29/0/0	29/0/0	29/0/0	29/0/0	29/0/0	29/0/0	28/0/1

**Table 6:** Comparison of ablation experiment results.

Algorithm		OOA	OOA1	OOA2	OOA3	OOA4	IOOA
F1	best	1.1072 × 10^2^	1.0160 × 10^2^	**1.0019 × 10^2^**	1.0055 × 10^2^	1.1358 × 10^2^	1.0064 × 10^2^
	std	2.0639 × 10^3^	3.8573 × 10^3^	2.5250 × 10^3^	3.6187 × 10^3^	3.0153 × 10^3^	**1.1831 × 10^3^**
	mean	1.6832 × 10^3^	3.2105 × 10^3^	1.8126 × 10^3^	2.7222 × 10^3^	2.4205 × 10^3^	**1.1867 × 10^3^**
F5	best	5.1890 × 10^2^	**5.1393 × 10^2^**	5.2487 × 10^2^	5.1592 × 10^2^	5.2487 × 10^2^	**5.1393 × 10^2^**
	std	1.1031 × 10^1^	1.0260 × 10^1^	1.3360 × 10^1^	**7.0837 × 10^0^**	1.1439 × 10^1^	7.6256 × 10^0^
	mean	5.4109 × 10^2^	5.3472 × 10^2^	5.4177 × 10^2^	5.3099 × 10^2^	5.3778 × 10^2^	**5.2839 × 10^2^**
F6	best	6.0213 × 10^2^	6.0314 × 10^2^	6.0315 × 10^2^	**6.0043 × 10^2^**	6.0264 × 10^2^	6.0054 × 10^2^
	std	1.6537 × 10^0^	1.5704 × 10^0^	1.6586 × 10^0^	9.6473 × 10^−1^	1.4716 × 10^0^	**7.9452 × 10^−1^**
	mean	6.0504 × 10^2^	6.0590 × 10^2^	6.0597 × 10^2^	6.0194 × 10^2^	6.0509 × 10^2^	**6.0190 × 10^2^**
F13	best	1.4333 × 10^3^	5.8351 × 10^3^	2.1592 × 10^3^	1.4516 × 10^3^	**1.4125 × 10^3^**	1.5640 × 10^3^
	std	1.1306 × 10^4^	9.9082 × 10^3^	1.3485 × 10^4^	1.1899 × 10^4^	1.2776 × 10^4^	**9.4116 × 10^3^**
	mean	1.8538 × 10^4^	1.6429 × 10^4^	1.9604 × 10^4^	1.6978 × 10^4^	1.7232 × 10^4^	**1.4177 × 10^4^**
F14	best	1.4547 × 10^3^	1.4714 × 10^3^	1.4706 × 10^3^	1.4653 × 10^3^	**1.4325 × 10^3^**	1.4726 × 10^3^
	std	1.1063 × 10^3^	4.7430 × 10^2^	7.2744 × 10^2^	5.0391 × 10^2^	8.7348 × 10^2^	**2.9734 × 10^2^**
	mean	2.0363 × 10^3^	1.9225 × 10^3^	1.9678 × 10^3^	1.7594 × 10^3^	1.9346 × 10^3^	**1.7263 × 10^3^**
F17	best	1.7366 × 10^3^	1.7318 × 10^3^	1.7376 × 10^3^	1.7267 × 10^3^	1.7346 × 10^3^	**1.7109 × 10^3^**
	std	1.5167 × 10^2^	1.1144 × 10^2^	1.4694 × 10^2^	9.8211 × 10^1^	1.2044 × 10^2^	**9.2822 × 10^1^**
	mean	1.9029 × 10^3^	1.8431 × 10^3^	1.8896 × 10^3^	1.7894 × 10^3^	1.8748 × 10^3^	**1.7879 × 10^3^**
F18	best	9.1530 × 10^3^	8.8868 × 10^3^	8.4757 × 10^3^	4.2022 × 10^3^	1.1162 × 10^4^	**3.0568 × 10^3^**
	std	1.3929 × 10^4^	1.2516 × 10^4^	1.3089 × 10^4^	1.3955 × 10^4^	1.3974 × 10^4^	**1.1592 × 10^4^**
	mean	2.7623 × 10^4^	2.5891 × 10^4^	2.8326 × 10^4^	2.0167 × 10^4^	3.0526 × 10^4^	**1.9815 × 10^4^**
F20	best	2.1543 × 10^3^	2.1568 × 10^3^	2.1535 × 10^3^	2.0375 × 10^3^	2.1399 × 10^3^	**2.0270 × 10^3^**
	std	1.6904 × 10^2^	1.4266 × 10^2^	1.1479 × 10^2^	1.1560 × 10^2^	1.5095 × 10^2^	**8.5300 × 10^1^**
	mean	2.3284 × 10^3^	2.3198 × 10^3^	2.3055 × 10^3^	2.2009 × 10^3^	2.3237 × 10^3^	**2.1846 × 10^3^**
F21	best	2.3187 × 10^3^	2.3157 × 10^3^	2.3176 × 10^3^	**2.3135 × 10^3^**	2.3175 × 10^3^	**2.3135 × 10^3^**
	std	9.0146 × 10^0^	1.0494 × 10^1^	9.3061 × 10^0^	9.0363 × 10^0^	8.6022 × 10^0^	**7.7997 × 10^0^**
	mean	2.3340 × 10^3^	2.3317 × 10^3^	2.3356 × 10^3^	2.3301 × 10^3^	2.3334 × 10^3^	**2.3291 × 10^3^**
F23	best	2.6623 × 10^3^	2.6691 × 10^3^	2.6697 × 10^3^	2.6566 × 10^3^	2.6688 × 10^3^	**2.6612 × 10^3^**
	std	1.1916 × 10^1^	9.8837 × 10^0^	1.3457 × 10^1^	1.2851 × 10^1^	1.4316 × 10^1^	**9.2988 × 10^0^**
	mean	2.6871 × 10^3^	2.6826 × 10^3^	2.6886 × 10^3^	2.6801 × 10^3^	2.6898 × 10^3^	**2.6776 × 10^3^**
F27	best	3.2132 × 10^3^	3.2204 × 10^3^	3.2153 × 10^3^	3.2109 × 10^3^	3.2220 × 10^3^	**3.1953 × 10^3^**
	std	1.3655 × 10^1^	1.1637 × 10^1^	1.4645 × 10^1^	1.2951 × 10^1^	1.4255 × 10^1^	**8.7317 × 10^0^**
	mean	3.2430 × 10^3^	3.2383 × 10^3^	3.2381 × 10^3^	3.2296 × 10^3^	3.2426 × 10^3^	**3.2200 × 10^3^**
F29	best	3.3706 × 10^3^	3.3641 × 10^3^	3.3698 × 10^3^	**3.3457 × 10^3^**	3.3887 × 10^3^	3.3538 × 10^3^
	std	1.3940 × 10^2^	1.5654 × 10^2^	1.5056 × 10^2^	9.6393 × 10^1^	1.7950 × 10^2^	**9.6361 × 10^1^**
	mean	3.5920 × 10^3^	3.5427 × 10^3^	3.5515 × 10^3^	3.4563 × 10^3^	3.5893 × 10^3^	**3.4434 × 10^3^**

The bold black data points indicate the best results within each set of experiments.

**Table 7:** Results of hyperparameter optimization for hyperspectral image classification.

	IOOA	OOA [17]	PSO [10]	GWO [11]	SSA [12]	AOA [13]	SCSO [14]
Best OA	0.976310	0.971635	0.974596	0.975713	0.974726	0.977791	0.975817
Worst OA	0.971531	0.971219	0.971271	0.971271	0.971220	0.436750	0.971271
Mean OA	0.973731	0.971453	0.973100	0.973271	0.972209	0.917232	0.972248
Std OA	0.001806	0.000172	0.001387	0.002028	0.001350	0.168881	0.001696
Avg ${log}_{10}C$	2.1323	3.3976	2.3968	2.5642	2.9403	1.4366	3.0378
Avg ${log}_{10}\gamma$	1.8314	2.4628	1.9676	2.0462	2.2411	1.7482	2.2852

**Table 8:** Results of the Wilcoxon rank-sum test on the Pavia University dataset.

	OOA [17]	PSO [10]	GWO [11]	SSA [12]	AOA [13]	SCSO [14]
*p* Value	0.00195313	0.55664063	0.64257813	0.01953125	0.01367188	0.16015625
+/NaN/−	+	NaN	NaN	+	+	NaN

**Table 9:** Comparison of representative methods on the IOOA and Pavia University datasets.

Method	Model Type	Training Protocol	OA (%) *
SMLR	Statistical classifier	10% training	78.78
KSMLR	Kernel statistical classifier	10% training	93.00
LELM	Extreme learning machine	10% training	91.23
2D-CNN	CNN-based classifier	10% training	96.63
3D-CNN	CNN-based classifier	10% training	96.34
M3D-CNN	CNN-based classifier	10% training	95.95
SVM+PCA-20	PCA+SVM	70% training	95.65
SVM+R-PCA-30	P-PCA+SVM	70% training	93.42
LightGBM+PCA-20	PCA+LightGBM	70% training	95.53
IOOA-SVM	Optimized SVM	10% training	97.37

* The literature results are collected from the corresponding published papers. The results of SMLR, KSMLR and LELM are from Cao et al.; the results of 2D-CNN, 3D-CNN, and M3D-CNN are from Roy et al.; and the results of SVM+PCA-20, SVM+R-PCA-30, and LightGBM+PCA-20 methods are from Ustuner. Since different studies may adopt different training/testing ratios, preprocessing procedures, feature extraction strategies, and validation protocols, this comparison is provided as a reference rather than a strictly identical experimental comparison.

## Data Availability

All data included in this study are available upon request by contacting the corresponding author.

## References

[1] Song Y., Zhang J., Liu Z., Xu Y., Quan S., Sun L., Bi J., Wang X. (2025). Deep Learning for Hyperspectral Image Classification: A Comprehensive Review and Future Predictions. Inf. Fusion.

[2] Ullah F., Ullah I., Khan R.U., Khan S., Khan K., Pau G. (2024). Conventional to Deep Ensemble Methods for Hyperspectral Image Classification: A Comprehensive Survey. IEEE J. Sel. Top. Appl. Earth Obs. Remote Sens..

[3] Melgani F., Bruzzone L. (2004). Classification of Hyperspectral Remote Sensing Images with Support Vector Machines. IEEE Trans. Geosci. Remote Sens..

[4] Rizkallah L.W. (2025). Optimizing SVM Hyperparameters for Satellite Imagery Classification Using Metaheuristic and Statistical Techniques. Int. J. Data Sci. Anal..

[5] Zito F., Talbi E.-G., Cavallaro C., Cutello V., Pavone M. (2025). Metaheuristics in Automated Machine Learning: Strategies for Optimization. Intell. Syst. Appl..

[6] Ding S., Chen L. Classification of Hyperspectral Remote Sensing Images with Support Vector Machines and Particle Swarm Optimization. Proceedings of the 2009 International Conference on Information Engineering and Computer Science (ICIECS 2009).

[7] Samadzadegan F., Hasani H. (2012). Determination Optimum SVMs Classifiers for Hyperspectral Imagery Based on Ant Colony Optimization. Key Eng. Mater..

[8] Zhao C., Zhao H., Wang G., Chen H. (2020). Improvement SVM Classification Performance of Hyperspectral Image Using Chaotic Sequences in Artificial Bee Colony. IEEE Access.

[9] Zhu X., Li N., Pan Y. (2019). Optimization Performance Comparison of Three Different Group Intelligence Algorithms on a SVM for Hyperspectral Imagery Classification. Remote Sens..

[10] Kennedy J., Eberhart R. Particle Swarm Optimization. Proceedings of the ICNN’95—International Conference on Neural Networks.

[11] Mirjalili S., Mirjalili S.M., Lewis A. (2014). Grey Wolf Optimizer. Adv. Eng. Softw..

[12] Mirjalili S., Gandomi A.H., Mirjalili S.Z., Saremi S., Faris H., Mirjalili S.M. (2017). Salp Swarm Algorithm: A Bio-Inspired Optimizer for Engineering Design Problems. Adv. Eng. Softw..

[13] Abualigah L., Diabat A., Mirjalili S., Abd Elaziz M., Gandomi A.H. (2021). The Arithmetic Optimization Algorithm. Comput. Methods Appl. Mech. Eng..

[14] Seyyedabbasi A., Kiani F. (2023). Sand Cat Swarm Optimization: A Nature-Inspired Algorithm to Solve Global Optimization Problems. Eng. Comput..

[15] Storn R., Price K. (1997). Differential Evolution—A Simple and Efficient Heuristic for Global Optimization over Continuous Spaces. J. Glob. Optim..

[16] Tanabe R., Fukunaga A.S. Improving the Search Performance of SHADE Using Linear Population Size Reduction. Proceedings of the 2014 IEEE Congress on Evolutionary Computation (CEC).

[17] Wang K., Abualigah L., Smerat A., Li J., Wu X., Liu H., Shao Z., Mirjalili S. (2026). A Nature Recoil Mechanism-Based Octopus Optimization Algorithm for Solving the Global and Constraint Optimization from Engineering Structural Design Problems. J. Comput. Des. Eng..

[18] Zhang D., Wang Z., Sun F. (2024). Somersault Foraging and Elite Opposition-Based Learning Dung Beetle Optimization Algorithm. Appl. Sci..

[19] Xu Q., Meng Z. (2025). Differential Evolution with Multi-Stage Parameter Adaptation and Diversity Enhancement Mechanism for Numerical Optimization. Swarm Evol. Comput..

[20] El-Shorbagy M.A., Ahmed A.A. (2025). Studying Complex Nonlinear Systems of Equations by Modified Beluga Whale Optimization Algorithm with Analysis. Results Eng..

[21] Li J., Meng Z. (2024). Elite-Guided Resampling and Multi-Mutation-Based Differential Evolution with Exponential Crossover for Numerical Optimization. Expert Syst. Appl..

[22] Chen Z., Fu B., Yang Y. (2025). A Novel Elite-Guided Hybrid Metaheuristic Algorithm for Efficient Feature Selection. Biomimetics.

[23] Fan X., Wang H., Zhuo Z., Bei S., Li Y., Liu Z. (2026). A Novel Dung Beetle Optimization Algorithm Based on Lévy Flight and Triangle Walk. Future Gener. Comput. Syst..

[24] Qin Y., Deng L., Li C., Zhang L. (2024). CIR-DE: A Chaotic Individual Regeneration Mechanism for Solving the Stagnation Problem in Differential Evolution. Swarm Evol. Comput..

[25] Lin S., Xu H. (2025). An Adaptive Differential Evolution Algorithm Based on Adaptive Evolution Strategy and Diversity Enhancement. Results Eng..

[26] Biedrzycki R., Arabas J., Jagodziński D. (2019). Bound Constraints Handling in Differential Evolution: An Experimental Study. Swarm Evol. Comput..

[27] Okwuashi O., Ndehedehe C.E., Olayinka D.N., Akpomrere R., Eyo E., Ogbijara U. (2025). Testing the Suitability of v-Support Vector Machine for Hyperspectral Image Classification. Int. J. Image Data Fusion.

[28] Guo Y., Wang Q., Hu B., Qian X., Ye H. (2025). Two-Stage Unsupervised Hyperspectral Band Selection Based on Deep Reinforcement Learning. Remote Sens..

[29] Wainer J., Fonseca P. (2021). How to Tune the RBF SVM Hyperparameters? An Empirical Evaluation of 18 Search Algorithms. Artif. Intell. Rev..

[30] Lopez E., Gorla G., Etxebarria-Elezgarai J., Amigo J.M., Seifert A. (2026). The Importance of Choosing a Proper Validation Strategy in Predictive Models. Part 2: Recipes for (Avoiding) Overfitting—A Tutorial. Anal. Chim. Acta.

[31] Zhang J., Sanderson A.C. (2009). JADE: Adaptive Differential Evolution with Optional External Archive. IEEE Trans. Evol. Comput..

[32] Yang Q., Chu S.-C., Pan J.-S., Chou J.-H., Watada J. (2024). Dynamic Multi-Strategy Integrated Differential Evolution Algorithm Based on Reinforcement Learning for Optimization Problems. Complex Intell. Syst..

[33] Liu J., Feng J., Yang S., Zhang H., Liu S. (2024). Dynamic ε-Multilevel Hierarchy Constraint Optimization with Adaptive Boundary Constraint Handling Technology. Appl. Soft Comput..

[34] Ahmad M., Distefano S., Khan A.M., Mazzara M., Li C., Li H., Aryal J., Ding Y., Vivone G., Hong D. (2025). A Comprehensive Survey for Hyperspectral Image Classification: The Evolution from Conventional to Transformers and Mamba Models. Neurocomputing.

[35] Won J., Lee H.S., Lee J.W. (2025). A Review on Multi-Fidelity Hyperparameter Optimization in Machine Learning. ICT Express.

[36] Hyperspectral Remote Sensing Scenes. https://www.ehu.eus/ccwintco/index.php/Hyperspectral_Remote_Sensing_Scenes.

[37] Cao F., Yang Z., Ren J., Jiang M., Ling W.-K. (2017). Linear vs. Nonlinear Extreme Learning Machine for Spectral-Spatial Classification of Hyperspectral Images. Sensors.

[38] Roy S.K., Krishna G., Dubey S.R., Chaudhuri B.B. (2020). HybridSN: Exploring 3-D–2-D CNN Feature Hierarchy for Hyperspectral Image Classification. IEEE Geosci. Remote Sens. Lett..

[39] Ustuner M. Randomized Principal Component Analysis for Hyperspectral Image Classification. Proceedings of the 2024 IEEE Mediterranean and Middle-East Geoscience and Remote Sensing Symposium (M2GARSS).

